# Map-based cloning of *QFhb.mgb-2A* identifies a *WAK2* gene responsible for Fusarium Head Blight resistance in wheat

**DOI:** 10.1038/s41598-019-43334-z

**Published:** 2019-05-06

**Authors:** Agata Gadaleta, Pasqualina Colasuonno, Stefania Lucia Giove, Antonio Blanco, Angelica Giancaspro

**Affiliations:** Department of Environmental and Territorial Sciences (DiSAAT), University of Bari “Aldo Moro”, Via G. Amendola 165/A - 70126, Bari, Italy

**Keywords:** Biotic, Environmental impact

## Abstract

*Fusarium graminearum* is one of the most threating pathogen of wheat, responsible for *Fusarium* head blight (FHB) which annually leads to yield losses, grain quality decay and accumulation of harmful mycotoxins in kernels. Host resistance represents the most effective approach to limit disease damages; however, only a limited number of resistant loci have currently been detected in durum genotypes. In this work we report the map-based cloning of a FHB-QTL on 2A chromosome of durum wheat, introgressed from a resistant line derived from the Chinese wheat cv. Sumai-3. A marker enrichment of the QTL region was carried out leading to the inclusion of 27 new SNPs respect to the previous map. A wall-associated receptor-like kinase (*WAK2*) gene was identified in the region and sequenced, in the resistant parent (^R^P) one gene was predicted accounting for a genomic sequence of 5,613 structured into 6 exons, whereas two adjacent genes were predicted on the same DNA plus strand of the susceptible parent (^S^P).t The involvement of *WAK2 gene* in FHB resistance mechanism was assessed by gene expression comparison between resistant and susceptible wheat lines, and disease symptoms evaluation in 3 TILLING mutants for WAK protein function.

## Introduction

*Fusarium* head blight (FHB, scab), prevalently caused by the hemibiotrophic fungus *Fusarium graminearum* (teleomorph *Gibberella zeae* Schwabe), is one of the most widespread and devastating crop disease affecting small grain cereals in temperate regions of the world^1^. In durum and bread wheat, FHB is annually responsible for serious economic threats, accounting for huge losses in grain yield and a substantial decay in quality of starch and storage proteins^2^ due to kernel contamination with harmful mycotoxins. Among the most abundant toxins, deoxynivalenol (DON) and zearalenone (ZEA) accumulate in wheat heads leading to pinky, shriveled grains unsuitable for human or animal feeding. In order to ensure consumers’ health, the European Union (EU) and other countries have set the maximum allowed levels of mycotoxins in wheat and wheat-derived food stuffs (1.75 ppm in unprocessed durum wheat; 0.75 ppm in pasta; 0.5 ppm in bread and bakery; 0.2 ppm in baby food; The European Commission, 2006). The large economic losses caused by scab represent a strong incentive to identify traits underlying FHB resistance, which could be the targets of future breeding programs aimed to obtain more productive and healthy varieties. As the conventional agrochemical practices are costly and only partly effective, breeding for host resistance represents the most efficient and environmentally sustainable strategy to manage FHB disease. Resistance mechanisms are classified as passive or active^3^. The first one is associated with morphological traits, such as plant height, awn length, spike density, ear compactness and heading date. Active mechanisms account for five different components: resistance to initial infection (Type I), resistance to fungal spread within the spike (Type II), resistance to kernel infection (Type III), tolerance (Type IV) and resistance to mycotoxin accumulation (Type V)^3,4^.

Although none of the wheat varieties is completely immune^5^, resistant lines prevalently carrying hallmarks of type-II resistance were more easily identified in hexaploid wheat (*Triticum aestivum L*.). Such examples are the Chinese cultivar Sumai-3 and its derivatives^6^, many Japanese accessions^5,7,8^, the Brazilian cultivar Frontana^7,9^ and some germplasm from Eastern Europe as “Prag 8”^10^. However, these lines are not always suitable for commercial purposes due to inappropriate agronomic traits^11^.

Compared to common wheat, durum varieties (*Triticum turgidum* ssp. *durum*) express a higher susceptibility to *FBH*, but missing of D-genome is not the main issue as most QTL have been mapped on A and B genome in hexaploid germplasm^11,12^. Durum vulnerability is also due to some morphological traits like spike compactness, anthers retention inside the floret and early flowering^13,14^, and it is actually increasing due to the spreading of durum acreages from the original Mediterranean area to more humid climates of Central Europe. For all these reasons, increasing yield and resistance is among the priority goals of modern breeders. Unfortunately, durum breeding for FHB resistance is still challenging due to the poorness of resistance sources in the tetraploid gene pool^15^ and to the necessity to incorporate resistance traits into already adapted germplasm. Durum wheat cultivars with FHB resistance comparable to that of common wheat have not yet been found^15–18^, but some genotypes can be exploited due to a moderate-to-high resistance which allows to limit the loss of production and accumulation of mycotoxins. At present, only partially tolerant accessions have been identified among wild ancestors or cultivated subspecies of *T. turgidum*^15^ such as ssp. *carthlicum*^19^ and ssp. *dicoccum*^18,20^, or durum landraces^16,18,21–23^. However, incorporation of resistance loci from such wild species into elite durum lines is time-consuming due to linkage drag^24^. Several attempts to introgress resistance from common into durum genotypes have been made, but this still remains a hard task because of the complex inheritance of hexaploid resistance into a durum background^20,25^. Some success in the use of hexaploid sources for the obtainment of durum wheat lines with high resistance levels, have been recently reported by Prat *et al*.^26^ and Giancaspro *et al*.^27^.

Resistance to *F. graminearum* is a quantitative trait with a polygenic inheritance influenced by the collective action of several QTL and genotype-by-environment interaction. By means of linkage analysis and genome-wide association study (GWAS), more than 200 QTL associated with various components of FBH resistance were detected on several chromosomes of common wheat, especially using recombinant inbred (RI) or doubled haploid lines^3,11,24,28–36^. In particular, the most effective QTL were derived from the resistant Chinese wheat accession “Sumai-3” or its close relatives, and were named *Fhb1* and *Fhb2*^30,31,37–40^ respectively located on 3B and 6B chromosome short arms accounting for type-II resistance, and *Qfhs.ifa.5A*^29,30^ responsible for type-I. Noteworthy, type-II resistance conferred by *Fhb1* on 3BS has been associated with the activity of conversion of DON into the less toxic form of DON-3-O-glucoside, through the enzymatic activity of a UDP-glucosyltransferase (UGT) gene^41,42^.

A smaller number of QTL have been detected in durum wheat, and most overlapped with loci detected in hexaploid wheat, implying a common genetic basis and a collinearity between hexaploid and tetraploid genotypes^20,43^. In particular, durum QTL detected on 3B^16,20^ and 6B^19,20^ coincided respectively with the major QTL *Fhb1* and *Fhb2* identified in common wheat, even if the effect of such loci in reducing FHB severity is smaller respect to those of common wheat^31,37^. Despite numerous QTL studies, only one QTL has been cloned by Rawat *et al*.^42^ so far, which reported a pore-forming toxin-like (PFT) gene located at *Fhb1* locus conferring FHB resistance. PFT is predicted to encode a chimeric lectin with two agglutinin domains and an ETX/MTX2 toxin domain.

A particular attention should be placed to homoeologous group 2 chromosomes: several FHB-QTL have been mapped on 2A and 2B with a R^2^ ranging from 3% to 27%^11,37,44–46^. A major QTL on 2A chromosome for both incidence and severity with an R^2^ of 12%, was found by Giancaspro *et al*.^27^: in this work for the first time, FHB resistance was successfully transferred from hexaploid to tetraploid wheat in a RIL population obtained from crossing an hexaploid line derived from the resistant Chinese cv. Sumai-3 and the susceptible durum cv. Saragolla. This locus co-localizes with a pectin methylesterase (*WheatPME-1*) gene physically mapped in 2BS1-0.53-0.75 and C-2AS5-0.78 bins^27,47^. Pectin content and methylesterification in grasses has largely been associated with plant resistance to several pathogens^48–52^.

The aim of the present work was the cloning of Q*Fhb.mgb-2A* and the identification of candidate genes putatively involved in the regulation of FHB resistance in durum wheat, that will facilitate to decipher the genetic basis of disease response and detect key traits to be efficiently transferred in practical breeding programs.

## Results and Discussion

### Marker enrichment of *QFhb.mgb-2A* region and QTL analysis

With the aim to construct a fine map of the *QFhb.mgb-2A* region, new SNP markers were surveyed from the wheat 90 K iSelect assay^53^ and included in the durum wheat 2A chromosome map previously developed^27^ in an 135 RIL population derived from 02-5B-318 (FHB-resistant, ^R^P) x Saragolla (FHB-susceptible, ^S^P). In this work, two major QTL (R^2^ = 12%) for FHB incidence and severity had been detected respectively on chromosome arms 2AS (closest marker IWB63138) and 2BS (closest marker IWB55365) Table 1. In particular, these QTL were found to co-localize with two *WheatPME-1* (pectin methylesterase) genes physically mapped on the short arms of chromosome group 2, respectively, in 2BS1-0.530.75 and C-2AS5-0.78 bins^47^. *PME* is a gene which modulates the degree of cell wall methyl-esterification, and is suggested to be involved in the response mechanism to *Fusarium* infection.

**Table 1:** QTL detected for FHB incidence and severity by Inclusive Composite Interval Mapping (ICIM) and Genome-wide Composite Interval Mapping (GCIM) performed on 2A chromosome map enriched with new SNP markers developed by Giancaspro *et al*.^27^.

Chromosome arm	Linkage group	Closest marker	Absolute map position (cM)	Bologna 2012			Bologna 2013		
				Add	LOD	R^2^	Add	LOD	R^2^
**Incidence**									
2 AS	2A-1	IWB63138	28.4	9.2	3.5	12	10.5	3.7	12
**Severity**									
2AS	2A-1	IWB63138	28.4	9.1	3.1	12	9.7	3.4	12

Add = Additive effect denoting the contribute of the resistant or susceptible line allele.

R^2^ = percentage of phenotypic variance explained by each QTL.

**Figure 1:**
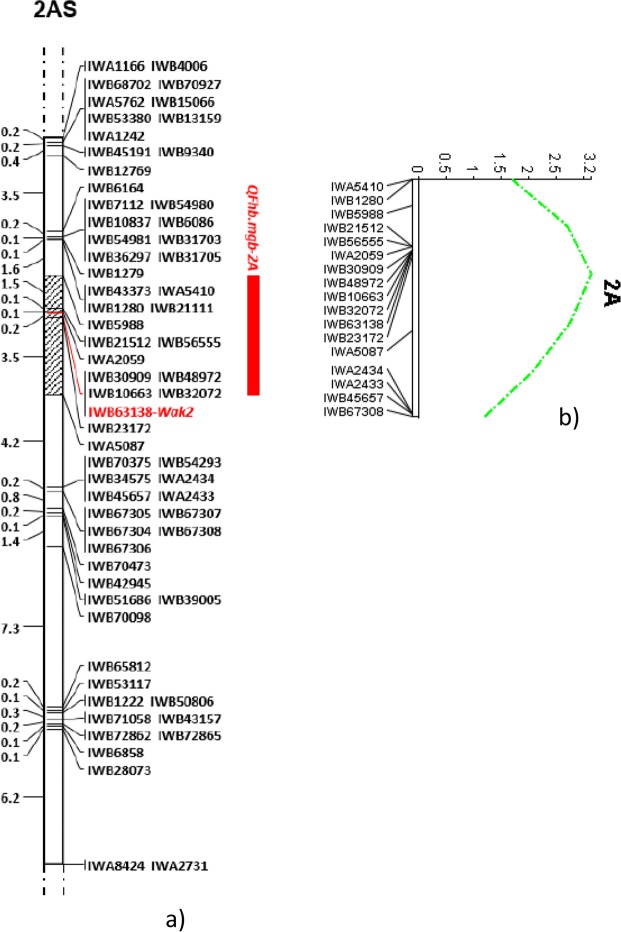
Saturation of durum wheat 2A chromosome region containing the FHB-QTL identified by Giancaspro *et al*.^27^. Map was obtained in the 135 RIL population derived from crossing the FHB-resistant bread wheat line 02-5B-318 and the FBH-susceptible durum cv. Saragolla. The grey bar indicates the peak region including 11 SNP markers between IWB5988 and IWA5087. The closest marker to *QFbh.mgb-2A* (IWB63138) is reported in bold red (**a**) and QTL LOD curve in green (**b**).

Following a new elaboration of the genetic map, a total of 65 SNP markers were re-localized on the linkage group containing the FHB-QTL, with 27 more markers with respect to the existing map, covering a total length of 32 cM with a SNP density of approximately 2 markers/cM (Fig. 1). Also the QTL mapping analyses was repeated, and identified a QTL region spanning over 5.3 cM, containing an overall number of 11 markers: the proximal and distant SNPs were represented by IWB5988 and IWA5087 respectively, and the closest marker was confirmed to be IWB63138 as in the previous published map with the same value of R^2^ 12%. Linkage analysis revealed a very high number of co-segregating SNP markers mapping on the same locus: in particular, redundancy (co-migrating loci mapping within 0.1 cM) was comprised between 2 and 9.

### Identification of candidate genes for FHB resistance in the *QFhb.mgb-2A* region

**Figure 2:**
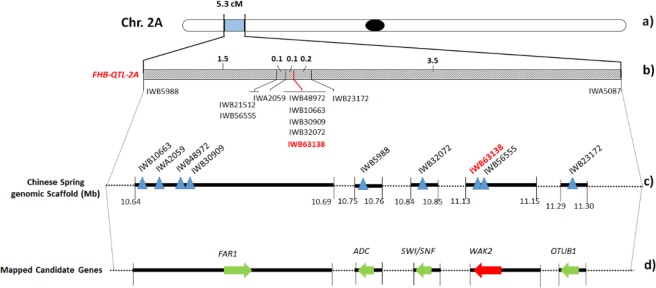
Fine mapping of *QFhb.mgb-2A* chromosome of durum wheat. (**a**) Chromosome location of the FHB-QTL on 2A short arm. (**b**) Genetic map of the QTL peak region of 5.3 cM containing 11 SNP markers. Closest marker is reported in bold red. (**c**) Physical map of QTL-associated SNPs (triangles) on 2A chromosome. Black rectangles represent the genomic scaffold of the reference cv. Chinese Spring with their respective position within the chromosome. (**d**) Candidate gene discovery. Arrows indicate ORFs identified in the QTL region. *FAR1* = Fatty Acyl-CoA Reductase; *WAK2* = Wall-associated receptor kinase 2; *ADC* = Arginine decarboxylase; *SMARCA3* = SWI/SNF-related matrix-associated actin-dependent regulator of chromatin subfamily A member 3; *OTUB* = Ubiquitin thioesterase otubain.

In order to identify candidate genes involved in the FHB resistance, a gene study was carried out by using the POTAGE tool for speeding up gene discovery in wheat. In detail, gene finding was carried out in the peak region of *QFhb.mgb-2A* comprised between IWB5988 and IWA5087, spanning over a length of 5.3 cM (Fig. 2). According to the PopSeq map, 29 unique genes were retrieved in the region which contained 9 mapped SNPs (Table S1). In particular, a first group of 4 SNP markers (IWA2059, IWB30909, IWB48972 and IWB10663) resulted localized within the *FAR1* (Fatty Acyl-CoA Reductase 1) gene, the 2 most associated markers (IWB56555 and IWB63138) were mapped in *WAK2* (Wall-associated receptor kinase 2), and IWB5988, IWB32072 and IWB23172 were detected within *ADC* (Arginine decarboxylase), *SMARCA3* (SWI/SNF-related matrix-associated actin-dependent regulator of chromatin subfamily A member 3) and *OTUB* (Ubiquitin thioesterase otubain) genes, respectively.

In order to physically anchor the 9 SNPs to chromosome 2A, their sequences were extended in the URGI database and localized onto three larger scaffolds (Fig. 2). Each scaffold contained the complete sequences of the candidate genes, with a length ranging from 5 kb of IWGSC_V3_chr2AS_scaffold_2430 (containing IWB32072), to 47 kb of IWGSC_V3_chr2AS_scaffold_1114 (containing IWA2059, IWB30909, IWB48972 and IWB10663). In particular, the scaffold containing the FHB-QTL closest marker IWB63138 (together with IWB56555) was 16,427 bp long (IWGSC_V3_chr2AS_scaffold_1426).

**Figure 3:**
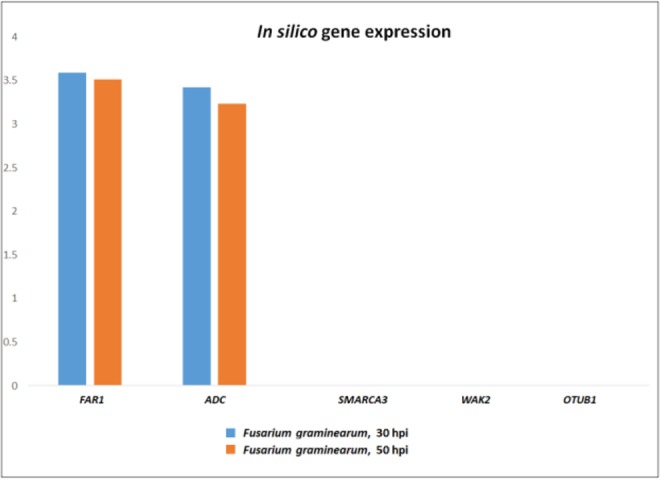
*In silico* gene expression of FHB candidate genes mapping on *QFhb.mgb-2A* region, at 30 and 50 hours post *F. graminearum* infection on spikes of the susceptible durum wheat cv Remus (from *expVIP database*). *FAR1* = Fatty Acyl-CoA Reductase; *WAK2* = Wall-associated receptor kinase 2; *ADC* = Arginine decarboxylase; *SMARCA*3 = SWI/SNF-related matrix-associated actin-dependent regulator of chromatin subfamily A member 3; *OTUB1* = Ubiquitin thioesterase otubain.

Details retrieved from POTAGE website are reported in Table S1, and include the functional annotation for all the putative candidate genes and their expression profile referred to the scale of Zadok^54^ (assessment of the developmental stages of wheat spike, with specific reference to anthesis (Zadok code 69) which is the growth stage more susceptible to *Fusarium* infection. This search identified the most expressed gene in spike in normal condition represented by *FAR1*, a group with medium values ranging from 8 to 1 FPKM, and 18 genes with a very low expression (<1). Notably, among the 10 genes annotated as “not expressed” (FPKM = 0), we found the wall-associated receptor kinase 2 (*WAK2*) containing the IWB63138 marker. To assess the involvement of such candidate genes in the FHB resistance mechanism and generate more biological insights, a comparative survey was carried out in the expVIP database^55^ using the SNPs mapping within the genes. The aim was the evaluation of gene expression variation following *Fusarium graminearum* infection of spikes, in particular at 30 and 50 hpi (the available dataset specifically referred to the susceptible durum wheat cv. Remus). This study confirmed the results of POTAGE site indicating a high expression level for *FAR1* and *ADC* genes, and a very low expression for *SMARCA3*, *OTUB* and *WAK2* (Fig. 3).

Among all the five candidate genes detected in the QTL region, we decided to deeply investigate *WAK2* first of all because a preliminary study of gene expression between the two parental lines identified the *WAK2* as the best candidate. Moreover, this gene was found co-localized with the QTL *QFhb.mgb-2A* (IWB63138), and it is referred to be involved in several pathogenicity mechanisms. WAKs are proteins allowing plant cells to respond to their external environment thanks to an extracellular region associated to the pectin fraction of cell wall^56–58^ and an intracellular Ser/Thr kinase domain. In crop plants WAKs show a very high affinity for CW pectin, binding both to long pectin polymers (involved in physiological processes requiring cell expansion) or to pectin fragments (OGs) released during pathogen attacks or environmental stresses, by forming networks with other receptor pathways. WAKs are involved in various plant functions like cell elongation, morphogenesis^59,60^ and protection against pathogens^61^. *WAK2* gene was chosen as a good candidate also because it could be related to a pectin methylesterase (*WheatPME-1*) gene which was previously mapped on the same region of 2A chromosome^47^, and has been assessed to be involved in the resistance mechanism to *F. graminearum* in wheat^51,62–65^.

### Isolation and characterization of *WAK2* (wall-associated kinase) gene in resistant and susceptible wheat lines

**Figure 4:**
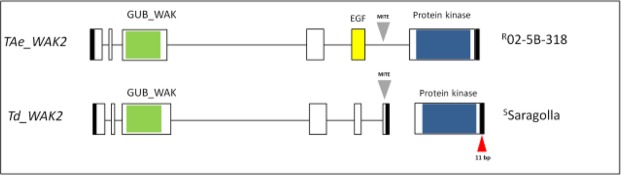
Gene structure and protein domain prediction of *WAK2* gene in the FHB-resistant hexaploid accession 02-5B-318 and the FHB-susceptible tetraploid cv. Saragolla. Exons are represented as rectangular boxes, and introns are black lines between the exons. Green: GUB_WAK: Galacturonan-binding wall-associated receptor kinase; yellow: EGF: Calcium-binding EGF (Epidermal Growth Factor)-like domain; blue: PROTEIN KINASE: Ser/Thr Kinase cytoplasmic catalytic domain; black boxes: 3′ and 5′ UTR. Grey arrow: MITE transposon. Red arrow: 11 bp nucleotide insertion.

With the aim to isolate the *QFhb.mgb-2A* region, we started from the most associated SNP marker identified in the QTL analysis (IWB63138). The sequence of 101 bp containing the SNP was extended in URGI database. Then, by using different primer combinations opportunely designed to cover the entire contig, the sequence on the A-genome was firstly isolated in the hexaploid 02-5B-318 accession (Fig. S1) and in the durum wheat cv. Saragolla (Fig. S2), and physically localized on 2A chromosome by using nulli-tetrasomic aneuploid lines. Sequence isolated on 2A chromosome and associated to IWB63138 was undergone a prediction of potential genes by using Softberry software (Fig. 4) and BLAST with wheat ESTs. In the resistant parent (02-5B-318, ^R^P) one gene was predicted accounting for a genomic sequence of 5,613 structured into 6 exons (mRNA of 2,262 bp), whereas two adjacent genes were predicted on the same DNA plus strand of the susceptible parent (Saragolla, ^S^P): the first one accounted for a gDNA sequence of 4,316 bp comprising 6 exons for a total mRNA length of 1,278 bp; the second gene was shorter and composed by one single exon of 966 bp. ^S^P and ^R^P shared the same length for the second and the fourth exon (47 and 248 bp), whereas dimensions of the other three exons (1, 3 and 5) were slightly different. The biggest difference between gene structure in the two parental lines consisted in the length of the last exon, which accounted for 960 bp in ^R^P and only 8 bp in ^S^P. Interestingly, the second gene predicted in Saragolla (966 bp) perfectly matched to the last exon of 02-5B-318.

Based on this sequence variation, a primer pair was opportunely designed to amplify a polymorphic fragment of 368 bp in 02-5B-318 or 373 bp in Saragolla, with could be used as a functional marker to employ in MAS breeding programs for FHB resistance *(WAK2-FHB-2A)*.

Sequence polymorphisms detected between ^R^P and ^S^P exerted important differences also in WAK protein structure (Fig. 4). *In silico* translation of 02-5B-318 mRNA resulted in an amino acidic sequence of 753 residues containing three functional domains: 1) Wall-Associated receptor kinase galacturonan-binding (GUB_WAK); 2) Calcium-binding EGF-like domain (EGF); 3) Ser/Thr kinase (catalitic domain). In contrast, the two proteins predicted in Saragolla were made respectively of 425 and 321 amino acids, the first one containing only the GUB_WAK domain and the second one including only the Ser/Thr kinase.

**Figure 5:**
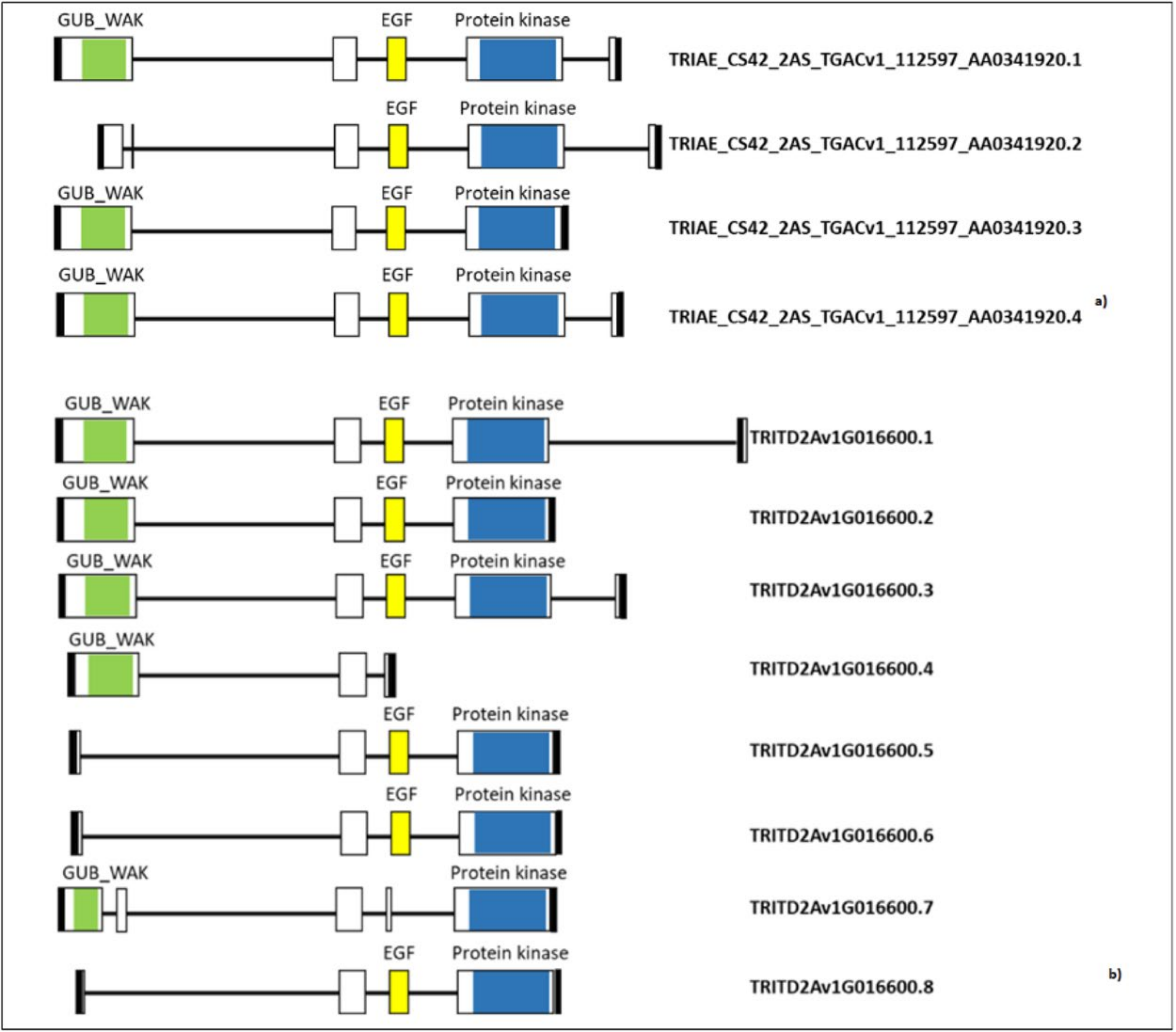
Splicing variants of *WAK2* gene. Predicted protein domains are in colors. Exons are represented as rectangular boxes, and introns are black lines between the exons. Green: Galacturonan-binding wall-associated receptor kinase (GUB_WAK); yellow: EGF: Calcium-binding EGF (Epidermal Growth Factor)-like domain; blue: PROTEIN KINASE: Ser/Thr Kinase cytoplasmic catalytic domain; black boxes: 3′ and 5′ UTR. (**a**) Splicing variants in *Triticum aestivum* (from EnsemblPlants database: https://plants.ensembl.org/index.html). (**b**) Splicing variants in *Triticum durum* (from Interomics cv Svevo genome browser: http://d-gbrowse.interomics.eu/gb2/gbrowse/Svevo/).

Sequences of *WAK2* gene were also obtained in Svevo and Chinese Spring reference varieties, by searching in Interomics and EnsemblPlants databases, respectively. This search confirmed the polymorphism found between ^S^P (identical sequence to Svevo) and ^R^P (identical sequence to Chinese Spring), and allowed to identify several transcript variants of *WAK2* corresponding to alternative splicing forms. In particular, 4 different transcripts were detected in Chinese Spring and 8 in Svevo (Fig. 5). In both durum and common wheat, the most variable regions of the transcripts were the first and the last exons, which influenced not only the gene size (984 to 2,325 bp in durum, and 1,827 to 2,277 bp in Chinese Spring) but also the presence of functional domains. The most variable domain in hexaploid wheat was the extracellular GUB_WAK, whereas variation in tetraploid wheat involved all the three domains.

Compared to dicotyledonous plants, the *WAK* gene family has considerably expanded in cereals; in fact, in such species genes are organized in very complex and wide families encountering for tens of members. More than 100 genes have been identified in the genomes of rice^66^ and maize^67^, while 26 have been counted in *Arabidopsis*^68,69^. Among the principal evolutionary mechanisms for the expansion of this gene family, it has been reported the insertion of retrotransposon sequences like MITE (Miniature Inverted-repeat Transposable Element) which can produce duplicated gene copies in tandem, breaking of the preexisting gene, or creation of alternative splicing sites^66^. For this reason, the genomic sequences of 02-5B-318 and Saragolla were launched in TREP database. Results showed that variation in gene size and structure between common and durum wheat could be due to the insertion of a 165 bp transposable MITE element which localized in the last exon of ^R^P and the last intron (exon 5–6) of ^S^P (Fig. S2). A search in EnsemblPlant database identified the same MITE element on several chromosomes of *Triticum aestivum* (2A, 2D, 3B and 4D) and *Hordeum vulgare* (1 H, 2 H and 3 H), highlighting its influence on the induction of alternative transcription mechanisms.

### Isolation of *WAK2* gene in FHB-resistant and FHB-susceptible wheat RILs

**Figure 6:**
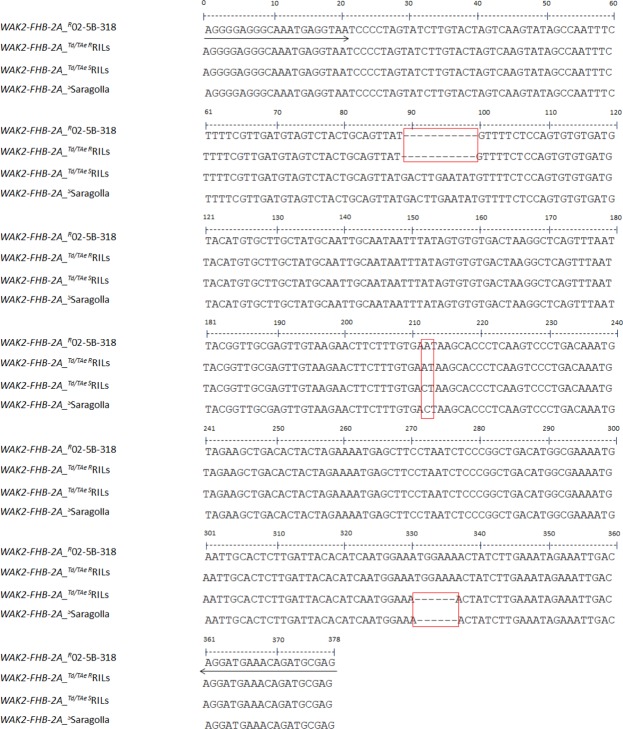
Sequence polymorphism of *WAK2* gene between resistant and susceptible wheat lines. Transversion and in/del are highlighted in red boxes. Arrows indicate primer sequences amplifying a polymorphic fragment of 368 bp in ^R^P/^R^RILs and 373 bp in ^S^P/^S^RILS to employ as functional marker.

In order to confirm the association of Saragolla and 02-5B-318 haplotypes respectively with FHB-susceptibility and resistance, sequencing of *WAK2* gene was obtained also in 5 FHB-resistant and 5 FHB-susceptible lines selected among the durum and bread wheat RILs obtained by the cross 02-5B-518 x Saragolla^47^. Interestingly, both durum and hexaploid ^R^RILs had the same nucleotide sequence of 02-5B-318, while tetraploid and hexaploid ^S^RILs were completely identical to Saragolla. These results were further confirmed by amplification of ^R^RILs and ^S^RILs gDNA with the abovementioned functional marker (*WAK2-FHB-2A*), which gave a 368 bp fragment in all the ^R^RILs and a 375 bp fragment in all the ^S^RILs (Fig. 6).

### Differential expression of *WAK2* candidate gene in FHB-resistant and FHB-susceptible wheat lines

The involvement of *WAK2* candidate gene in wheat response to *Fusarium* infection was evaluated by a time-course expression study in ^R^P and ^S^P, parents of a previously obtained RIL population segregating for *Fusarium* resistance^47^. Expression profiles of common and durum lines were compared at 24, 48 and 72 hours after infection. For each line the histogram shows the normalized expression relative to the mock inoculated controls (RNE, Relative Normalized Expression). At each time point, expression values are reported as the average of three biological replicates Comparative expression of *WAK2* candidate gene in 02-5B-318 (^R^P), Saragolla (^S^P), 5 resistant (^R^RILs) and 5 susceptible (^S^RILs) durum wheat progenies of the cross 02-5B-318 x Saragolla, registered at different times after *F. graminearu*m infection (24, 48, 72 hpi).

**Figure 7:**
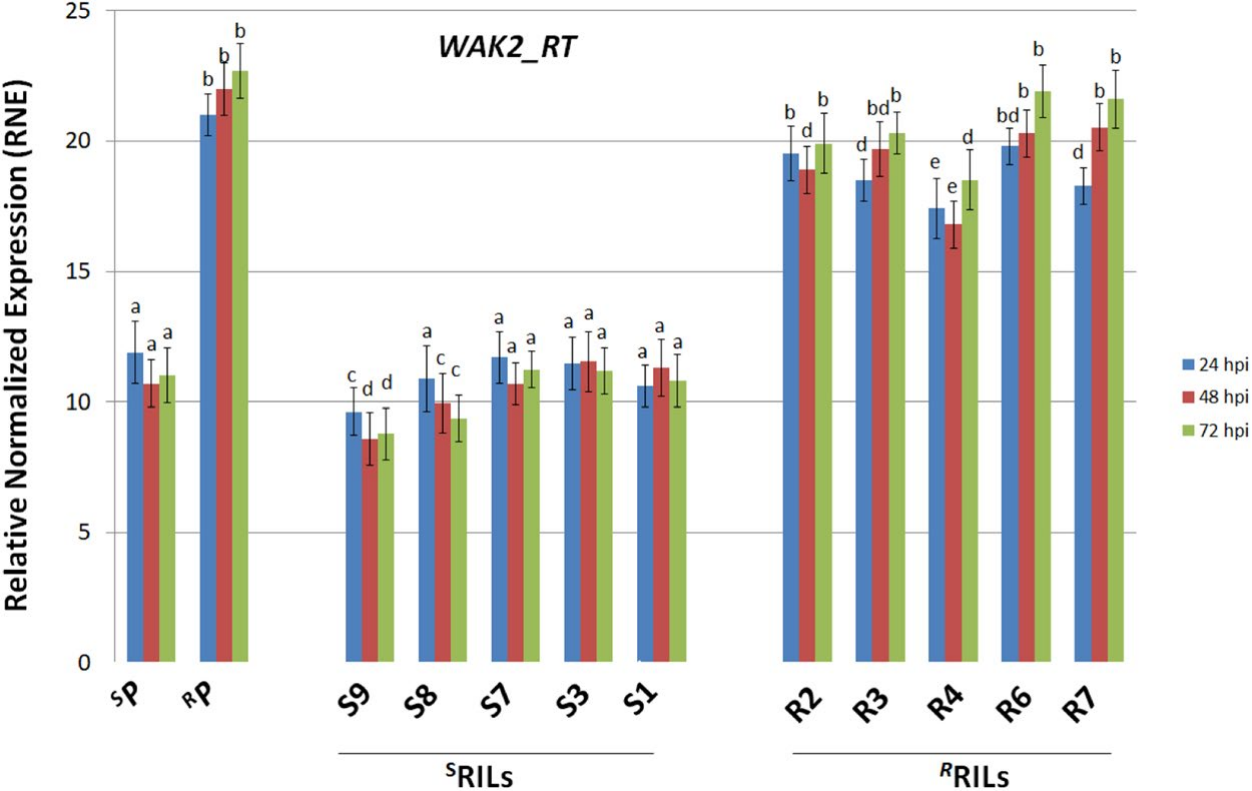
Comparative expression of *WAK2* candidate gene in 02-5B-318 (^R^P), Saragolla (^S^P), 5 resistant (^R^RILs) and 5 susceptible (^S^RILs) durum wheat progenies of the cross 02-5B-318 x Saragolla, registered at different times after *F. graminearu*m infection (24, 48, 72 hpi). For each line the histogram shows the normalized expression relative to the mock inoculated controls (RNE, Relative Normalized Expression). At each time point, expression values are reported as the average of three biological replicates (each run in triplicate) ± SD (vertical bars), normalized against the mean of three reference genes: ADP-ribosylation factor (*ADP-RF*), Cell Division Control protein (*CDC*), and RNase L inhibitor-like protein (*RLI*). Letters indicate datasets significantly different between parental lines and the two groups of RILs, according to analysis of variance (ANOVA) followed by Tukey’s test (p < 0.01). hpi = hours post infection.

As reported in Fig. 7, *WAK2* was expressed in both parents with significant differences which were more evident at 72 hpi, when abundance of gene transcripts in the ^R^P doubled that of the ^S^P (22.7 vs 11). Slight differences were instead observed in gene expression registered at different times after *F. graminearu*m infection (24, 48, 72 hpi). The observation that the gene was constitutively expressed in both resistant and susceptible lines for all the time course, could be due to the fact that besides response to pathogen infection, *WAK* gene family is involved in several other cellular pathways, including cellular expansion, germination or reaction to abiotic stresses^56,58^.

*WAK2* association with *Fusarium* infection was also assessed by measuring transcript accumulation in 10 durum wheat lines (Fig. 7), 5 selected among the most resistant (^R^RILs) and 5 among the most susceptible (^S^RILs) of the population obtained by crossing 02-5B-318 and Saragolla. Differences between the two groups reached the maximum level at 72 hpi, when the values of *WAK2* relative expression in ^R^RILs doubled that of the ^S^RILs; in particular, RNE values were comprised between 18.5 (R4) and 21.9 (R7) in the ^R^RILs, and between 8.8 (S9) and 11.2 (S7) in the ^S^RILs. The trend of the two groups of tetraploid lines reflected that of the two parents: in both ^R^RILs and ^S^RILs the expression level of *WAK2* did not show significant variations at the different time-points after infection: ^R^RILs showed a slight time-course increase of gene expression similar to 02-5B-318, whereas the level of gene transcript somewhat decreased in ^S^RILs like in the susceptible parent Saragolla.

**Figure 8:**
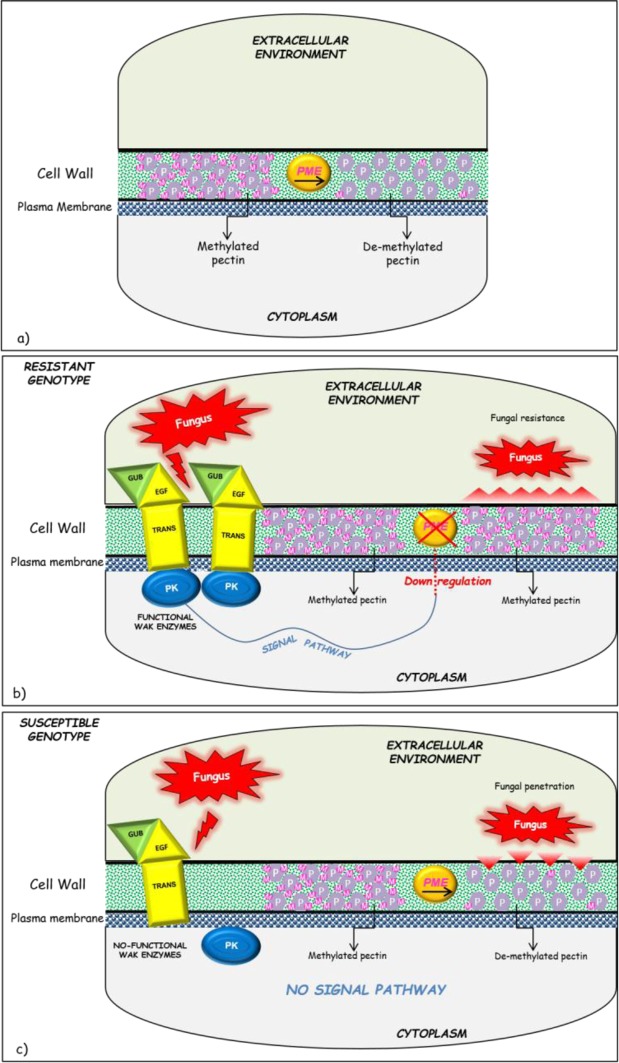
A model of interaction between *WAK2* and *PME-1* candidate genes in the mechanism of response to *Fusarium* infection in durum wheat. (**a**) Action of *PME-1* on plant cell wall (CW) pectin. (**b**) *WAK2*/*PME-1* interaction in resistant genotypes: the perception of fungal presence by WAK2 functional enzymes activate a cascade signal pathway which probably down-regulates *PME-1* gene, thus keeping CW pectin in a highly methylated status which protects plant cell against fungal penetration. (**c**) *WAK2*/*PME-1* interaction in susceptible genotypes: with the fungus, WAK2 no-functional enzymes do not activate the intracellular signal pathway. Consequently, *PME-1* gene is normally expressed thus leaving CW pectin less methylated and more susceptible to fungal infection.

The different expression pattern observed between ^R^P/^R^RILs and ^S^P/^S^RILs seemed to confirm the actual involvement of *WAK2* gene in the resistance mechanism, and reflects a different response to fungal attack exerted by resistant and susceptible genotypes. In the present work, we speculated a model (illustrated in Fig. 8) which takes into account also the role of a pectin methylesterase (*WheatPME-1*) gene, previously physically located near *WAK2* on wheat 2A chromosome, and already referred to be involved in FHB resistance mechanism^47,49,51,62,65^.

In detail, WAKs (wall-associated kinase) are receptor-like protein kinases binding to pectin fragments (OG, oligogalacturonans) generated by pathogens or wounding. These molecules account for a N-terminal galacturonan-binding extracellular domain (GUB_WAK) followed by a transmembrane domain, and a highly conserved kinase domain on the cytoplasmic side of plasma membrane. GUB_WAK is anchored to the membrane but does not contain any kinase activity. It serves to the recognition and binding of different molecules (ligands) released in the environment during a biotic or abiotic stress. The extracellular domain is composed by several sub-domains: for example, WAK is composed of one or several repeats of EGF (Epidermal Growth Factor) domain, which are located near the transmembrane domain. The cytoplasmic portion of the enzyme is the catalitic domain and is the site of the kinase activity. WAKs exist in several isoforms: the intracellular domains of the isoforms are well conserved, whereas the extracellular domains are less conserved and more variable. As reported in rice, *WAK* genes can be annotated either as kinase domain-containing protein genes or receptor-like protein (RLP) genes, depending on the presence of the kinase domain^66^.

WAK enzymes allow cells to recognize and respond to their extracellular environment by activating different signaling pathways leading to the repression or induction of downstream genes involved in stress response^56–58^. *PME* is a gene directly responsible for the de-methylation of cell wall pectin: the gene is induced in susceptible lines (making cell wall more porous to fungal penetration), or down-regulated in resistant lines (keeping pectin highly-methylated and less susceptible to degradation by fungal enzymes)^27,47^.

Based on the results of this study, we speculate that in the resistant plants *WAK2* gene is correctly expressed and produces functional CW receptors. As a consequence, the host plant cell is responsive to the fungus presence and may activate an intracellular signaling pathway which probably exerts a negative modulation of downstream *PME* genes. In this way, CW pectin remains in a highly methylated status and results more rigid and resistant to *Fusarium* penetration.

On the other hand, in the susceptible lines, two forms of WAK proteins are produced by the alternative splicing of *WAK2* gene (illustrated in Fig. 4): the first one lacks the Ser/Thr kinase cytoplasmic domain, and the second one misses the EGF-transmembrane domain. According to our hypothesis, these latter are not-functional enzymes which lead to a less efficient intracellular cascade signaling. As a consequence, the mechanism of *PME* down-regulation is slowed, so that cell wall pectin keeps highly de-methylated becoming more porous and susceptible to *Fusarium* infection. The behavior of *WheatPME-1* gene reported by Lionetti *et al*., and confirmed by Giancaspro *et al*. supported our hypothesis in fact they found a different gene expression profile with a gene down-regulation in resistant lines.

### Identification and analysis of TILLING mutants

TILLING mutants for *WheatPME-1* and *WAK2* genes were identified using the resource developed by exome sequencing of 1,535 Kronos mutants by the University of California Davis, and available on line (see Material and Methods) (Table 2). In this tetraploid wheat population, all the surveyed mutations were G-to-A or C-to-T transitions, as expected from alkylation by EMS (Ethyl Methane Sulfonate), and most of them were in heterozygous state. Most of nucleotide changes were located at the end of second and fourth exons, respectively for *PME-1* and *WAK2* genes, and were missense type (Table 2).

**Table 2:** Tetraploid mutants selected from a TILLING population of the durum wheat cv.

Gene	Genome	Mutant line	Genetic status	DNA effect	Protein effect^a^	PSSM^b^	SIFT^b^	Primer combination
*PME-1*	A	Kronos2396	homo	C1351T	Q455^*^	Stop codon		K9-K8
*PME-1*	B	Kronos2555	het	G1501A	R496K	—	—	K9-K8
*PME-1*	B	Kronos534	het	C1470T	P486S	8.5		K9-K8
*PME-1*	B	Kronos2131	het	C1437T	P475S	18.1	—	K9-K8
*WAK2*	A	Kronos3340	het	G5090A	M716I	12.5	0	K4-K5
*WAK2*	A	Kronos4209	het	G5046A	A702T	11.3	0	K4-K5
*WAK2*	A	Kronos3965	homo	G5010A	E690K	10.4	0.87	K4-K5

Kronos carrying mutations in candidate genes *PME-1* and *WAK2*.

^a^Protein coordinates are based on complete protein sequences from the corresponding genome in cv. Chinese Spring.

^b^PSSM = Position-Specific Scoring Matrix; SIFT = Sort Intolerant From Tolerant. PSSM and SIFT scores are not reported for mutations that cause premature stop codons or severe effects. PSSM > 10 or SIFT > 0.5 mean missense change dramatic for protein function.

For *PME-1* gene (A and B genomes), the optimal target region suggested by CODDLE was the end of the first exon, while the best mutations detected on the website fell in second exon. In particular, twenty-six total mutations were retrieved and were so distributed: twenty-two were located into the intron regions and did not produce any amino acidic change, one was detected on A genome and affected a premature stop codon (Kronos2396), one on B genome (Kronos2131) was predicted to be tolerated with high PSSM score (>10), and two for B genome (Kronos2555 and Kronos534) were missense.

For *WAK2* gene, CODDLE highlighted the fourth exon as an important region to estimate the expected proportion of the different types of mutations to be recovered. In fact, eleven mutations were totally detected on A genome using the on-line platform, exactly into exon 4: two mutations were found in the intron region between exon 3 and 4, six were silent changes and three (Kronos3340, Kronos4209 and Kronos3965) reported significant PSSM (>10) and SIFT scores (<0.05) with a predictable loss-of-function.

**Figure 9:**
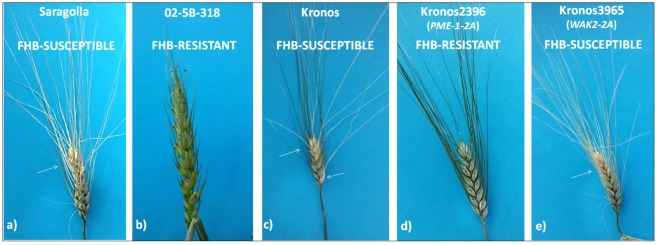
Pathogenicity assay conducted on TILLING lines of the durum wheat variety Kronos carrying mutation in *PME-1* and *WAK2* genes: ^R^Kronos2396 (premature stop codon) and ^S^Kronos 3965 (loss-of-function mutation). Artificial infection was carried out also on control lines ^S^Kronos, ^S^Saragolla and ^R^02-5B-318. Symptoms were evaluated 21 days after inoculation under growth chamber controlled conditions. White arrows indicate bleached spike areas showing FHB symptoms.

Seven mutants, four for *PME-1* and three for *WAK2* (Table 2), were taken into account since they were predicted to affect protein function, and tested for response to *Fusarium* infection. Phenotypic evaluation was conducted after inoculating mutant lines and controls (Kronos, Saragolla and 02-5B-318) under growth chamber controlled conditions. FHB symptoms were evaluated at 15 and 21 days after inoculation by measuring the percentage of infected spike area. As expected, the susceptible durum varieties Saragolla and Kronos showed severe disease symptoms accounting for a gravity of 70–80%, whereas the resistant hexaploid line 02-5B-318 expressed only a limited gravity of 5% affecting the acropetal spikelets (Fig. 9).

Among the four *PME-1* mutants, line Kronos2396 carrying a premature stop codon manifested a good resistance to *Fusarium* infection with only 5% of spikelets showing disease symptoms, while the other three mutants expressed a complete susceptibility. For what concerns mutants in *WAK2* gene, all the three lines carrying a predicted loss-of-function mutation (Kronos3340, Kronos4209, Kronos3965) showed severe disease symptoms. In particular, Fig. 9 reports the example of line Kronos3965 which clearly shows an extensive whitening along the whole spike and awns, containing shriveled gray kernels.

The examination of TILLING mutants carried out in the present study contributed to confirm the involvement of *PME-1* genes in the mechanism of wheat response to *Fusarium* infection. A mutation in pectin methylesterase gene leads to a highly methylated state of cell wall pectin, and consequently to a more compact CW allowing plant cell to oppose fungal penetration. On the other hand, a loss of function of *WAK2* receptor protein confirmed by sequences analysis of the gene in cv Kronos, causes an alteration of intracellular signally pathways acting on downstream stress response genes (probably on the down regulation of *PME-1*), thus making plant cell more susceptible to fungal attack.

In the future, *PME-1* mutations could be combined to develop double mutants whit a final stronger effect on phenotype; moreover, since *PME-1* and *WAK2* are both localized on 2AS chromosome, their mutations could be evenly combined within the same line. This approach could be very useful to furtherly validate the role of each gene and clarify their interaction in the resistance mechanism. Thanks to the development of a new functional marker within the *WAK2* gene, this work provides a powerful tool to improve and speed up future MAS breeding programs for FHB resistance in wheat.

## Methods

### Plant material

A markers enrichment of the *QFhb.mgb-2A* region was conducted in a 135 durum wheat Recombinant Inbred Line (RIL) mapping population previously developed at DISAAT (Department of Environmental and Territorial Sciences, University of Bari, Italy)^27,47^ by crossing the FHB-resistant bread wheat accession 02-5B-318 (^R^P, a breeding line derived from the resistant Chinese cv. Sumai-3) and the FHB-susceptible durum wheat cv. Saragolla (^S^P).

Molecular studies were carried out on ^R^P and ^S^P, plus a set of 5 susceptible (S) and 5 resistant (R) wheat lines selected among the tetraploid and the hexaploid populations obtained by crossing 02-5B-318 and Saragolla.

Wheat lines carrying mutations in two candidate genes (*WAK2* and *PME-1*) identified in *QFhb.mgb-2A* region were selected from a TILLING (*Targeted Induced Local Lesions In Genome*) population of the durum wheat variety “Kronos” developed by Dubcovsky: https://dubcovskylab.ucdavis.edu/wheat-tilling. Mutant lines for *PME-1* were: Kronos2396 (A genome) and Kronos2131, Kronos2555, Kronos534 (B genome). Mutants lines for *WAK2* were: Kronos3340, Kronos4209 and Kronos3965 (A genome) (Table 2).

### Map construction

The genetic map of durum wheat 2A chromosome previously developed by Giancaspro *et al*.^27^ was further saturated with additional SNP markers surveyed from the total 81,587 SNP sequences spotted on the Illumina wheat 90 K iSelect SNP assay (Illumina CSProR, SanDiego, CA, USA)^53,70^. Genotyping of the 135 RIL mapping lines was performed as described in Giancaspro *et al*.^27^.

Linkage analysis between markers and determination of the new linear order of loci were performed by JoinMap software v. 4.0^71^, using the regression mapping algorithm and the Kosambi mapping function to calculate map distances^72^. Linkage groups were established using the independence LOD parameter with a score of >10. Physically mapped SNPs^73,74^ were used as anchor loci and for physically allocate linkage groups on 2A chromosome.

### QTL detection

QTL detection was carried out using RIL means for individual environments described by Giancaspro *et al*.^27^, as well as means across environments. FHB-QTL mapping was re-performed with the Inclusive Composite Interval Mapping (ICIM) method^75^ using the QGene 4.0 software^76^ and using genome-wide composite interval mapping (GCIM) with the QTL.gCIMapping.GUI software^77^. Putative QTL were considered significant when markers were associated with the trait (FHB incidence and severity) at a LOD ≥ 3. The proportion of phenotypic variance explained by the QTL was determined by the square of the partial correlation coefficient (R^2^). Graphical representation of linkage groups was carried out using MapChart 2.2 software^78^.

### Identification of candidate genes for FHB resistance and *in silico* analysis

Wheat candidate genes (CGs) for FHB resistance were identified in the *QFhb.mgb-2A* region using an *in silico* approach. Sequences of SNP markers spanning the QTL region for 5.3 cM (from IWB5988 to IWA5087) were used to precisely fix the physical position of *QFhb.mgb-2A* along the 2A chromosome, and survey any predicted gene inside the region.

CGs were quickly identified using data publically available at *POPSEQ Ordered Triticum aestivum Gene Expression database* (POTAGE, http://crobiad.agwine.adelaide.edu.au/potage ^79^, reporting expression values in terms of fragments per kilobase of transcript per million fragments mapped (FPKM) using RNA-Seq reads and Cufflinks version 2.1.1^79^. All the identified candidate genes were screened through a BLASTn search (http://blast.ncbi.nlm.nih.gov/Blast.cgi) to confirm the SNPs mapped in the QTL.

Sequences of all SNP markers inside the region were taken into account and extended in URGI database (https://urgi.versailles.inra.fr/blast/blast.php) to retrieve scaffolds assignable to the A genome.

### Cloning of *QFhb.mgb-2A* sequence and *WAK2* gene characterization in durum and common wheat

Sequencing of the *QFhb.mgb-2A* region including the tightly associated SNP marker IWB63138 on contig IWGSC_V3_chr2AS_scaffold_1426, was conducted in the bread wheat accession 02-5B-318 and in the durum wheat cv. Saragolla, plus a set of 5 FHB-resistant and 5 FHB-susceptible lines selected among the durum and common wheat RIL populations obtained by crossing 02-5B-38 and Saragolla, and previously characterized for resistance and cell wall composition by^47,65^. Genomic DNA was isolated from all the lines as described by Gadaleta *et al*.^73^ starting from 0.1 gr of fresh leaves, then checked for quality and concentration at a Nanodrop device (Thermo Scientific, Walthman, MA, USA).

Ten different primer combinations were opportunely designed on the aforementioned contig by OligoExplorer (http://www.genelink.com/tools/gl-oe.asp), and used to PCR-isolate the QTL sequence.

Based on sequence variation of *WAK2* gene between Saragolla and 02-5B-318, a primer pair was opportunely designed to amplify a polymorphic fragment in length between the two lines, which could be used as an SSR functional marker to employ in MAS breeding programs for FHB resistance

*WAK2-FHB-2A_For*: 5′ AGGGGAGGGCAAATGAGGTAA 3′

*WAK2-FHB-2A_Rev*: 5′ CAGGATGAAACAGATGCGAG 3′

Amplification reactions were performed in final volumes of 10 µl containing: 1 µl of gDNA (50 ng/µl), 0.5 µl of 10 µM forward primer, 0.5 µl of 10 µM reverse primer, 1 µl of 200 µM dNTP mix, 2 µl of Buffer, 4.88 µl of ddH_2_O and 0.12 µl of *Phusion™ High-Fidelity DNA polymerase* (Thermo Fisher). Reactions were run in *MyCycler™ Personal Thermal Cyclers* (Bio-Rad®) according to the following *touch-down* profile: 98 °C for 30″ followed by 10 touchdown cycles of 10″ at 98 °C, 30″ at 65 °C (0.5 °C lower per cycle) and 1′ at 72 °C, followed by 25 cycles of 10″ at 98 °C, 30″ at 60 °C and 1′ at 72 °C, with a final extension step of 7 min at 72 °C. Amplification products were firstly checked for the expected molecular size by visualization on 1.5–2% agarose gels, then cleaned using ExoSAP-IT™ (USB, Cleveland, OH, USA) according to manufacturer’s instructions, and finally sequenced using the BigDye® Terminator Cycle Sequencing Kit (Applied Biosystems, Foster City, CA, USA) on a ABI-3500 Genetic Analyzer (Applied Biosystems, Foster City, CA, USA). Sequence alignments between the hexaploid 02-5B-318 accession, the durum wheat cv. Saragolla and the RILs were carried out using ClustalOmega (http://www.ebi.ac.uk/Tools/msa/clustalo/) and CodonCode Aligner software (CodonCode Corporation, Centerville, MA, USA).

All gene and protein structures were predicted by Softberry software (http://linux1.softberry.com) and validated by launching genomic sequences in EnsemblPlants database (https://plants.ensembl.org/index.html) for *Triticum aestivum*, and in Interomics cv Svevo genome browser (http://d-gbrowse.interomics.eu/gb2/gbrowse/Svevo/) for *Triticum turgidum*.

The genomic sequences corresponding to *WAK2* genes in durum and common wheat were screened for the presence of any transposable elements at the TREP database (TRansposable Elements Platform, http://botserv2.uzh.ch/kelldata/trep-db/blast/blastTREP.html).

Proteins were functionally characterized with the NCBI conserved domain search tool (www.ncbi.nlm.nih.gov/Structure/cdd/wrpsb.cgi).

### RNA isolation, cDNA synthesis and qRT-PCR experiments

Total RNA was extracted from spikes of resistant and susceptible plants of durum and bread wheat at 0, 24, 48 and 72 hours post infection with the *RNeasy Plant Mini Kit* (Qiagen®), checked on 1.5% denaturing agarose gel, then reverse-transcribed into double stranded cDNA through the *iScript cDNA Kit* (Bio-Rad) as described by Giancaspro *et al*.^65^. Primer sequences for Real-Time study of *WAK2* candidate gene were designed to amplify a fragment between 100 to 200 bp (*WAK2_RT_For:* 5′GGTATCGTGCTACTGGAGCTC3′; *WAK2_RT_Rev*: 5′CTCCCATGGTAGGCCTGTTA3′). After optimization of annealing temperature and primer concentration, qRT-PCR reactions were run in a CFX96 *Real-time System* (Biorad) following this thermal profile: 95 °C for 3′, followed by 40 cycles of: 95 °C for 10″ and 60 °C for 30″. *CDC* (Cell Division Control), *ADP-RF* (ADP-Ribosilation Factor) and *RLI* (RNase L Inhibitor-like protein) genes, were used as internal references to normalize *FHB-QTL-2A* expression data analysis were conducted as suggested in the work by Marcotuli *et al*.^80^. For each experiment 1 µl of a 1:20 cDNA dilution was used in a final volume of 10 µl containing 5 µl of SsoFast SybrGreen® SuperMix 10X (Bio-Rad), 2 µl of primer mix and 2 µl of *RNase*-free ddH_2_O. Reaction efficiency was calculated for both target and reference genes by running six-point standard curves of three-fold serial dilutions of cDNA in the same amplification plate of the samples. NT (No Template) and NA (No Amplification) were simultaneously amplified as controls. Each sample was run in triplicate. Data analyses were performed with the CFX ManagerTM 3.1 software, using the Normalized Expression mode (ΔΔCq) which calculated the relative quantity of target (*WAK2*) normalized to the relative quantity of internal references (geometric mean of the three reference genes). For both target and reference genes, relative expression was calculated as fold-change respect to the mock-inoculated controls at each harvesting stage, and determining the standard deviation (SD) for the relative quantity. All the results were analyzed by ANOVA (GenStat 14, v.18,VSN International Ltd, Hemel Hempstead, UK).

### Analysis of TILLING mutants and pathogenicity assay

Mutants for pectin methylesterase (*PME-1*) and wall-associated kinase (*WAK2*) candidate genes were selected from a TILLING population of the durum wheat Kronos variety available on the on-line platform: https://dubcovskylab.ucdavis.edu/wheat-tilling. Mutations were searched in the gene sequences provided by Lionetti *et al*.^47^ for *PME-1*, or obtained in the present study for *WAK2*. The bioinformatics tool for displaying and analyzing nucleotide polymorphisms PARSESNP (Project Aligned Related Sequences and Evaluate SNPs) available on line at: http://www.proweb.org/parsesnp/parsesnp_help.html ^81^ was used to determine the effect of single nucleotide polymorphisms (SNPs) on protein, based on the alignment of related proteins with the use of PSSM and SIFT. Mutations resulting in a premature stop codon, loss of splicing sites or with a potential disruptive effect of amino acid substitutions on the protein function, were given priority for the study of their effect on the final phenotype. Plants carrying the targeted mutations in a homozygous status were selected by sequencing with genome-specific primers combinations:

*WheatPME-1-B*_*For*:5′ATCATGGTACGCAATGCACT3′

*WheatPME-1-B*_*Rev*:5′CGCCCATGGAAGGAGTACTC3′

*WAK2-A_For:5*′AGAAGTTCTTACAACTCGCAACC3′

*WAK2-A_Rev*:5′AAAGAAGGCTAGGTACGGGG3′

The 02-5B-318 resistant line, the susceptible durum cultivars Saragolla and Kronos and all the TILLING mutants were tested for *Fusarium* resistance by performing artificial inoculation under controlled conditions. Spikes were sprayed at anthesis with a macroconidia suspension (about 10^6^ conidia/ml) of *F. graminearum* isolate PH-1 grown as described by Lionetti *et al*.^47^, then covered overnight with polyethylene bags and kept in the growth chamber with 80% humidity until FHB symptoms development. Symptoms were visually scored after 15 and 21 days as the percentage of infected spike area (severity, type-II resistance) according to the scale of Parry^82^.

## Supplementary information

Supplementary Information
