# Supplementary material for: Map-based cloning of QFhb.mgb-2A identifies a WAK2 gene responsible for Fusarium Head Blight resistance in wheat
Source: Sci Rep. 2019 May 6;9:6929. doi: 10.1038/s41598-019-43334-z (PMC6502796; doi:10.1038/s41598-019-43334-z)
Supplement: Supplementary file 1 — Supplementary Information [file 41598_2019_43334_MOESM1_ESM.pdf]

Title

Map-based cloning of *QFhb.mgb-2A* identifies a *WAK2* gene responsible for Fusarium Head Blight resistance in wheat

Agata Gadaleta[*†], *Pasqualina Colasuonno*[†], *Stefania Lucia Giove*, *Antonio Blanco*, *Angelica Giancaspro*

[†] These authors contributed equally to this work

Supplementary Figure S1 Genomic sequence of *QFhb.mgb-2A* region isolated in the FBH-resistant common wheat accession 02-5B-318 (^{R}P), containing the *WAK2* gene. mRNA and protein sequence are also reported.

Grey box: exons. Sequence in the box: transposable MITE element (165 bp). Green box: 6 bp insertion. Yellow: SNP

> *QFhb.mgb-2A_02-5B-318*, gDNA

GCACAGCGTCCTCCATATGGTCCTTGCAAACACACAAGTCAGCAAAATGTGGTCAAGTG
TGTCCTCCTCCTGTGTACAGAACAGGCAGGCGTCCTGGTGTGCTAATCCTCGTTGGGCA
AGTCGATCTGAGGTCCAGCATATGTTCTCCATGGCGAGCCAAAAGAAGAAACAACATC
GTAGCGGATCCCTCGATTTCCACGTGAATTCCACGGTTGGCACCACCTCTCTCCCTACAA
ACTTGGCGGCGTATGCAGATCTAACCGAAAACTGACTATCGTTTTCCCCTGACCACATA
ATGGTGTTTGTAGCTCCTTCATCGAGTTCAACTATGTCCACCATTTGCCATACTCCCATG
TACTCTTGTAGTGCGTCAAACTCCAAGTTTGGGCCAACATCCATCGTCCAAGTTTGGGCC
AACGTCCATCGTCCAGGTTCCCCTAGTCAATGCCTGTGGACAGTTCGAGATGTTTTGGC
GCGCTGCGGGATTTTAACATAGAGCGCCGGTGCAAGTTCCTCTAAATGTGTGTTATGTT
GTATATCATCTCTTCAGACAATCCAACAAATAGGTAATTAAATTTATACGCGCTATCTA
GTTAGACTCGCATTAACTTGACAAAAGTAAGATTTAGCGTATTAATGCTTGGCCTATTA
AAAAGATTTATACTAGTCCTTGAGATGGTGAGTATATAAGTGGCATGTGGCAAAGTAAT
ATACGCATAGTGAGGGATGACCACAGGAACTGGCCTAAGAAACACGCATATATGTCAT
CATATATATGTCATCTTGTTGAGCTCATGTCACCATAGCTTTGAAAGGGACACAAATTAT
GCATCGTTAATTATTGAGCTTGTGTGCTAGCTCAGAGACAACCACGGGGAGTTTGGGAC
TAGCCTAATACACAAACATATATGTCATCGCACATACGTCATGTTATTGTGCTTATGTCA
CCACAATTTGGAAAGTGACATAGACTGTGCGTCCTTATAATTACTGGGTTTGTGTGCTA
ACCCATCCACCAACCATTCAATTTGCATCAAATAAGTAAGTAATGATGTCAATTTGTCA
CACGGCCCATGTCGATCACCATCAACTCAAAAAGAAGCAGCAAACTATGTTATCGCAAC
ATTCCTTTGTGGAAAAAGAATCTTCAAACGACAATGCCATCAACAGAACGTGGAGCATA
TGAAAGTGAAACACAATCAAGGTATCTTTTCTGGGTTTTTTTAAGGCAAAAGTATCTTTT
CTGGTGATTTGCCAACGCCACACTATCCAGGTGGGCCGTCAGGGCTACGACCATCACCT
CTGTCGATGATTGGTACAGTAAGTATGTTGAGCAAGATAAACGAGCATCAGGTCGACAC
AGCAGGGTGCCTGGCGAGTGTTGTGCAATAGAAGTCGTAGTTCGACCAAACTGTGGCCA
TTGGTCACTCAACTTTCATGTCAGTCAGTTTGTGTTACTATATATATATCGGTTATGGGT
ATCAGAGGAGGCCGTGCGTCCAAAGCAATTTAGCCATGACACTGCGACAAACCCTCCTC
CTCCTACTTGTCTTCCATGCCTCGCTCGCCACGACCGCCGAGTTTGGCGAGCCGTTTCCG
CCCCCAGCCCCGGCGTCGCCTGAGCCAAAAAACTGTCCTGCCAAGTGCGGGGAAATAG
ACATCCCCTACCCATTCGGCGTCGGCGCTGGATGCTCCCTGTCGGGCCGCTTCGTCCTCA
CCTGCAACGAGATGACGAGTCCTCCAACCCTGCTCACCGGCACAGTCAAGGTCGCCAGC
ATCACGCTGGAGACGGCGCAGATGGTGGTCAACACCTACCTGACCTATAGCTGCGATGT
GCCGACCAGCAACACTACGATCAACCACACGAGCACCGATATGGCCCTTAACGTCGTCA
CCCCGTTCCTGCTCTCGCCATCCGAGAACGTGTTCACGGCCGTCGGGTGCAGCTCGACG
GCGAGGATCAAGGGCCGCGGCGCGAGCCCCTACCTCACTGGCTGCATCACGACCTGTGC
CAGGGTGAACGACACCGGTGACGACGGCACACCCTGCAGCGGGCACGGCTGCTGCCAG
GCCTCGCTGACTCCTAACCTCACCCAAGTCAGCGTGGAGTGGGAGAACAGGGGAGGCA
GAAGTCCTGTGGCTGAAAACCTGTGCCAGTATGCTTTCGTCGCTACCAAAGGCTGGTAC
GTGAAGAAATTCCTTTTCCCCTTTTTCCCCTTTCAAATCATCGTATACAGAATAACTTGG
CAATTAACCCACCTTCCTATAACGTCCTAGTAAGGTCTATGGGTTTCACTTTCACTGAGA
TTTTGATGAATTTCATTCAATTTCAATACACAGAAATATTTACCTGTTGGTCATATTTCA
GTGATTTGTGTTGTGCATAGGAATTCAAATATTTTTCTAAATATTTTTGAACTTTTTCAA

ACATTTTTTTAATTCAAGTGAACATTTCGGTCCTTTGGCCAAAATGCAAACCGAAATCAC
TGAACTTCAGTTATTTCAGTAGGTGCTAAACTGTTTCTGAAACTGAAATTTAAAACCAA
CGAGTACTACTACTGATTATAACTTGGCAATTGCTGACAGAACATATCATCGAAACAAA
CATGTTTTTTCGAAAAGGAGGATTACCTCTGATCTTTGCATCAAGCGATGTACACAATC
ATTTGTTTATTAAGGGAAACGATTCCCTACGACCGACCGACCGAAGCTTTGGCCAGTCG
GGTCGCTCCCGACCCACGCGTCGCACACCCACCAATGCTAGTGTGCCACGCGTCCCTAT
GTTCTTGTGCAATGCTAAAAAGGCACCAAACGGCGACGGCTCCCAGCACTGACCGGAG
CGGGATGTTGCAACCATCAAAAATACGAGAGCATACCTAAACACTTACCCTATTATTTG
ACTGCCATCCAAATTGGTTGTATGTAGTACATGTTACCGTCTCACATCGGTTGCATCCAA
AGGCCATAAGTTCCCTCACGTCTGCATGGTGTAGTAATGACCACGTATGGATCGATTCA
AACGATCGGTAGATAACCTGCAAAGAGTTTGTTGTAATGTTTGTTATGTTAAACACACA
ATCATTCCGATAGCTGCAAATAGCCCAAAGTAATGCACAAGCTCTGACTCGAATTCTTG
CCTTTGTATTGGACTTAACCCTATCTAACTAGCTACTACCAAACCGGATTAGGGATACTA
TTAGGTGGAGGTATGTTAAAGGACACATGTATTATTCGTCATAATAACTTGGCTAGTGG
GCATATAAGAAATAAGTGTTGAATCATTTTCTCGTGGTCACAGAAATAATGGTGTTTAT
TCCCTTCCCAACTACGTTTTGCCAAATTGTACTTTGTTAGAATCACGCTCTTATGAAAAA
AACTCATGAAAATCTTGATCTTTAAAGGCACTTTCGTTATCCAGAAATGAATAGACAGT
GTGTTCCAGTATTAATCAAGTCGACATACATAGATTTAACGAAAAACAAGTCTGAAACA
GAAAGAGCCCTTCAGAATGTATCGGGCTCATTGACAAGTTGACCTCCATCAAGCTTTGC
ATTAAATGCAGCCAGTGATCCAATCTTTCCCCAATCAGTGTTCATCTGAACTGTACATTT
AGAGGAACGATCCCAATATTGTACTCACAGATTCGTCCTTATGATGTACGATATTATAA
AAGCGTTAGATAATACTATAAGGCTAGAGGTGTCTCTCCCAGCCAAATATCCTCCCAAA
ATCTAATTGTACGCCCATCTCCAATAATAAACTTGCCCCTATTAAAGAAGATGACCTTAT
TTCGCATCAAAGCCTTCCAAAAGGGTGAATCGTTTGGCTAGGCCATTACCTGCGAGAAG
GTCTTGGAGTGTAAATGCTTGTTTCAAAACAACATGTAACCACTAACCATAGGATCTAG
TTTAGAAGATCTTGTCGCAACAAACCAATGGTGAGAAAAAAAGATCCTCGAACAGATTT
ATGGTTGGTGCTTAATGTAAGAATATAGTGAATCTTAGTGATATCATGCTTGCACACGA
GAGAGATGGGGTGGTGTTGCACGGTATGGTCAAGATGCATCGCAGCATCCTAGCCTGGT
CCATCATTCTACCAAAGTTATAGGTTTTATGAAGATAATGGCATGTTTTTAGTCTAGACA
TATGTACAAACGCTTCAAGTTGCAACAATCTTCCCAAAAAAAAAGTTGCAACAATCTTCA
CATATACTAATGGTTGAATTTGTCGAGCAGGTACAGTTTCAGAAAAAATGACCTGATTG
GGAATATGACATTCGCCAATAGACTTGGAAGCGGCCCTGTTGTTCCAGTTGTTCTTGACT
GGGCAGTTAGGGATGGAACATGCCCCCCGACTCTGGAGGGTAACGGCAAGGAAATTGT
TCCTAACGGTGCCGCCTGTGTTAGCAGTGAAAGCTATTGTGTAAATGCAAGTCATGGAG
CACCGGGCTATTTCTGCAATTGCTCCAAGGGATACACCGGTAATCCATACAAAAAAAAT
GGGTGCACAAGTATGTAAACTTCACTCCACTTGTTTGTTATTGCATATATATTCCTCTCG
CCTCAGAGTAATTTGAGATCTTTTTAATTGTATAGCACACCCTGGATACCGAGGAGGAC
AATTTGACCTTGGATAAGATTAGGGATCCCAGGATTTTTAGTGCTCGCTCACTATTTTTA
ACATTAATAAACTTGGAAGTAAATAATAGTATGATACTAATGTAAGACTTACATATTTC
ATCCCTTGTGGCCTTATTAATTCAAAGTCAGGATCTACTACAGCGGTACGGCCTTCCTTC
TACAAAAATGATATTG**G**TTGGTGTCTTTACTTTCATGCACTAAACTGAACAATGTGCAA
TGTTTTTCGTACATTTTTCAGATATTGATGAGTGCGCATTACGACGATCACCAAATTCCA
CAATGTACAAGAATATCTATCCTTGCCGTGGAGGGACATGCCACGATACCGAAGGTGAT
TATGAGTGTAAGTGTAATTTCGGACGAAGAGGAGACGGCAAGAGTGACAAAGGTTGTG
AACCCGTATTGTCCAGACCTGCAGT**T**GCAGTGATAGGTGAGGTATTGTATATATGCTCT
ACTTTCCTAAACTTCATGACAAAGCAGTCACCATTCCAACAACTTGCATCATATTCTTAT
TTCAAAAAAAAAACTTACATCATGTTCTTATATCACTGAATATCTATACCAAATTACTAG
TACATGTCATCAGGTGAAGTTGTGAACAGTTATGACTGTCTCTTAATATATACTCCCTCC
GTTCCTAAATATTTGTCTTTTTAGAGATTTCAAATGGACTACCACATACGGATGTATATA
GACATATTTTAAACTGTAGATTCACTCATTTTGCTCCGTATGTAGTCACTTGTTAAAATC
TCTAGAAAGATAAATATTTAGGAATGGAGGGAGTAGTTCGCATGACACCATTAACATG
CATATTTTTCGAAAAGGAATGAAAACCCTTGGCCCTGATGCACACAGCCTTTTGCATGA
ACATGCATGACCAAATCTTCTCTTGGGTACATGTCATGCAGTTACTTGGAGCAAGCTAG
TACT**T**GTCATATGTATGAACTCTCATGTTAGTGCTCGATCTCTGGCCATCTTGAAAAAAA

ATGTAACTACTTATATATGTCATCTGCTTGCAATTGCATGCAGGAACAATTGG**T**GCAAT
CGCATTACTGTTCGTGCTAGTAATATTTTTGCACATGGAGCGAGAGAACAGGAAGCTGA
GGGACCGATTCAACAGGAACGGCGGGTCGTTCCTCAAGAGCGCCGGGATCGAGATCTT
TACCAAGGACAAGCTGGGTCGCATCACAAACAAGTACAGCTGCATCATTGGCAAAGGT
GCCTTCGGTAAGGTCTACAAGGGGACCACCGACACCGGCGTTGTTGTTGCTGTGAAGCG
CTCCATCATCGTCAACGAGGACCGGCAGAAGGATTTCGCGAATGAGATCACGGTCCAAT
CCAAGATCAGCCACCCAAACCTGGTCCGGCTCGTGGGTTGCTGCCTGGAGACGGAGGTG
CCCATGTTGGTCTATGAGTTCGTCCCCAGAGGGAGCCTTCAGGACGTGCTTCACGGAAA
CAACCACCCTCTCCCACTGGAAACACGCCTCGACATTGCTATCAGCTCCTCTGAGGGGC
TCGATTACATGCACTCGCAGAGCCAGATGGTCCTCCACGGTGATGTCAAATCTGGCAAC
ATCCTCATCGACGACAGCTTCACGCCCAAGGTGTCGGACTTTGGGACGTCCAGGCTCAT
GTGCATCGACAAGGACCACACCAACTGGGTGGTCGGGAACAGCAGCTACATTGACCCC
GTGTACATGAAGACCGGGCTGCTCACCGCTAAGAGCGACGTGTACAGCTTTGGTATCGT
GCTACTGGAGCTCGTTACCCGAAAGAAGGCTAGGTACGGGGAGAA**T**CGTAGCCTTCCG
ATGGACTACGTCAAGGCTTCCAAGGATGGCACGGCAAGGCAGCTGTTTGATGAGGAGG
TTTCATCCAATGTTGAGGAGAACATGGAGTGCCTCGAGGACGTTGGCAAGATTGCAGTG
CAATGTCTCGAGGAAGACGTGAATAACAGGCCTACCATGGGAGAAGTCAGGAAGGAAC
TTGAAAAGTGTAAGACACAATGGTTGCGAAGCCAGGGGAGGGCAAATGAGGTAATCCC
CTAGTATCTTGTACTAGTCAAGTATAGCCAATTTCTTTTCGTTGATGTAGTCTACTGCAG
TTATGTTTTCTCCAGTGTGTGATGTACATGTGCTTGCTATGCAATTGCAATAATTTATAG
TGTGTGACTAAGGCTCAGTTTAATTACGGTTGCGAGTTGTAAGAACTTCTTTGTGAATAA
GCACCCTCAAGTCCCTGACAAATGTAGAAGCTGACACTACTAGAAAATGAGCTTCCTAA
TCTCCCGGCTGACATGGCGAAAATGAATTGCACTCTTGATTACACATCAATGGAAATGG
AAAACTATCTTGAAATAGAAATTGACAGGATGAAACAGATGCGAGTGTATAGACATTC
AAAATCAAAACATTTGGGTATGAAACTAGAGGTGGGAAGGGAACACTAAGCTGGATGA
ATGCAGGAAATGGAGATGCTCTGTACGTGCGCCGTCCAGCTGCTTCGTCTGCATTACAA
CAAGGACAATTAACAACTGAATCAGGTTCATACCATGCATACATGCATTAACCTGTTAA
GAGTCAGCAGCAACTAGGGGCGTCTACCTGTATCTGCAGGTTCAGCAAGTAGGGGCCTC
TATGTGCCCGGGTTTCCTTTTCCTCGCTCCGGCATCTGAAATTCCCTTCCTTGGCAAATG
CGCCACAGACCACAGCTACGGAACAAGTGCAACTGATTGGTTGACCACACAGCCGCGG
TCGCGCAATGCGCCGAGCACCTTGTGGGCGTGGTGGACATGCGGCCGCCCGGCCTGGCT
GCATCGCTGCTGGAGCAACGGTGTCGCGGTTCCGGGAAGGCAGATAACTAGCGAAGGT
TGCCAAGGACGACGGCGATGAGCCGGCACAGGTGTGATGTGGCCAGACGGCGGAAGCC
GGCGGCGGTCTGGGACGCCGGTGCGCGATGACAAGTCTCGCTAGAGGACAAGTCGGGC
CACCGATGGTTGTGCAGCCCACGGCCATTTTGACTGGGCCGCCCATTTGACGAACACGG
CGAGCTCCTCGTTGACAGCTACTGTAGCTTTGCAGTTTGTCTCAAAAAAAGCTACTGTA
GCGTGCTTGTCTCAAAAAAAAATCTACTGTAGCGTACGAAGTACTATAGCTCGCAGGTG
CACTGTAGCTTAAAAACAAAAACTTGCAGGTTTTACTGTAGCTGCGTTCAAAAAATGCA
ACCAACTTTTGAAAAAGTGGACTGTTTGATATACAAACAATTTTTCGAAATTTTACAAC
AAGTTTTGAATTTCGAATATTTCTTGAATACTTTGAACAATATTTGAATTTTTTGCAATA
TTTTTGCAAAACACGAAATTTTTTGGAAAACATTATCAAGTTTTTTGAAATTCTGAAGCA
TTTTTGAATATTTTTATTAATTTTTTAGAAAAGTGAACATTTTAAAGGAAAGCATGAACC
AAAATTTGAAATTTGTAGCATTTTATGGAAAGCGAACATTTTCCAAAATTCTGAACAAG
TTTTGAAGGCATTTTTCAAGAATTTATGAAAATAAAAAAGACAAATAGACGCAAAAAA
AACATAAAAGGAAAAAAAAAGGGAAACCGAAACAAAAATGGAAAACTAGAAAAAGCAG
TAAAGAAAATGACAGAAAAAAGAAAACCGGTTCAGGATCCTTCTAAAAGATTCTCAAA
ACCGATTGAAAAATATCCTCTCCTTCATCCCTTTTCTCTGCGGCAAACACGGTCTCCCTC
CCCTCTCTCAGCGCGCGCGCCACACCATCCCCTCGAGGCTTTGACAGGGTGAGGGGTGG
TTTCAGGCTTCGTTCTTATGCGGTTGGTGGTTGGCGTGGCGTTGTAGCTAGGTGAGTATT
GGATCGCATCTCTAATCTGTCTCACCCATTTTTGTAGGTCAACAACCCGAACTTCCTACA
CTGTACTATGTATACACATGCTAATACTTCTTCTGTGTTGGAGGTACTTGCTCTTCTTGCT
TGCTAGGATGATAGGATGATGCCATGCATGTGAGATACCAATTAAGAAGGGCAATAAT
TTACTACTACTTGTTATTATGTGTGCTAAATATATGGCATGCCAAACTATATCAGCCAAT
CATCAGCCTTGTAAGTTGTAGCGTTAAATAGATTATATTATTTGCAGGGCCTTTTTTTGG
GAAAAATATAACCTATGCGGCATCAGTCAACGCTTCATTTGGCTGTTTCAGACTGGCCT

GTGTTAATCCACTTATCCAAATGTGCACAAAAATAAAAAATCATACTTACAACCCAACT
GACACTCCTCTCCATACTTTAAGGGCCTCCAATCCATTTTACTCTTCTTTATTAGATACG
GCTGGGCTCTAATACACACGGAATATTTGAGTTCTCAAAAGTTTGCCTTCTCTCGACAG
AAGATGTTCCAAGGTCTTAAGTGGCTAAATCTTGCCTTTTGCAATTAGAGATATGGCAT
GATGTGTGCTACGCTGATATTTTTTGTTGAATGATCCTTTGTAATCTTGCTACTCTAACTT
TCTTGTTTCGCTGACTCTATTACGGCAAAATAAACCAAGGTTGTTTCTTGACTGTCTTGT
GTATGAAGAAACATCAACAATGATAACAAAATATGGATGGGTGCAGATACGATTGACG
GTATAAAGTTTGAACTCTCATTATGC

>*WAK2*_02-5B-318, mRNA, 2,262 bp
ATGTCGATCACCATCAACTCAAAAAGAAGCAGCAAACTATGTTATCGCAACATTCCTT
TGTGGAAAAAGAATCTTCAAACGACAATGCCATCAACAGAACGTGGAGCATATGAAA
GTGGGCCGTCAGGGCTACGACCATCACCTCTGTCGATGATTGGTACAAGGAGGCCGTG
CGTCCAAAGCAATTTAGCCATGACACTGCGACAAACCCTCCTCCTCCTACTTGTCTTCC
ATGCCTCGCTCGCCACGACCGCCGAGTTTGGCGAGCCGTTTCCGCCCCCAGCCCCGGC
GTCGCCTGAGCCAAAAAACTGTCCTGCCAAGTGCGGGGAAATAGACATCCCCTACCCA
TTCGGCGTCGGCGCTGGATGCTCCCTGTCGGGCCGCTTCGTCCTCACCTGCAACGAGAT
GACGAGTCCTCCAACCCTGCTCACCGGCACAGTCAAGGTCGCCAGCATCACGCTGGAG
ACGGCGCAGATGGTGGTCAACACCTACCTGACCTATAGCTGCGATGTGCCGACCAGCA
ACACTACGATCAACCACACGAGCACCGATATGGCCCTTAACGTCGTCACCCCGTTCCT
GCTCTCGCCATCCGAGAACGTGTTCACGGCCGTCGGGTGCAGCTCGACGGCGAGGATC
AAGGGCCGCGGCGCGAGCCCCTACCTCACTGGCTGCATCACGACCTGTGCCAGGGTGA
ACGACACCGGTGACGACGGCACACCCTGCAGCGGGCACGGCTGCTGCCAGGCCTCGC
TGACTCCTAACCTCACCCAAGTCAGCGTGGAGTGGGAGAACAGGGGAGGCAGAAGTC
CTGTGGCTGAAAACCTGTGCCAGTATGCTTTCGTCGCTACCAAAGGCTGGTACAGTTTC
AGAAAAAATGACCTGATTGGGAATATGACATTCGCCAATAGACTTGGAAGCGGCCCT
GTTGTTCCAGTTGTTCTTGACTGGGCAGTTAGGGATGGAACATGCCCCCCGACTCTGG
AGGGTAACGGCAAGGAAATTGTTCCTAACGGTGCCGCCTGTGTTAGCAGTGAAAGCTA
TTGTGTAAATGCAAGTCATGGAGCACCGGGCTATTTCTGCAATTGCTCCAAGGGATAC
ACCGATATTGATGAGTGCGCATTACGACGATCACCAAATTCCACAATGTACAAGAATA
TCTATCCTTGCCGTGGAGGGACATGCCACGATACCGAAGGTGATTATGAGTGTAAGTG
TAATTTCGGACGAAGAGGAGACGGCAAGAGTGACAAAGGTTGTGAACCCGTATTGTC
CAGACCTGCAGTTGCAGTGATAGGTGAGCGAGAGAACAGGAAGCTGAGGGACCGATT
CAACAGGAACGGCGGGTCGTTCCTCAAGAGCGCCGGGATCGAGATCTTTACCAAGGA
CAAGCTGGGTCGCATCACAAACAAGTACAGCTGCATCATTGGCAAAGGTGCCTTCGGT
AAGGTCTACAAGGGGACCACCGACACCGGCGTTGTTGTTGCTGTGAAGCGCTCCATCA
TCGTCAACGAGGACCGGCAGAAGGATTTCGCGAATGAGATCACGGTCCAATCCAAGA
TCAGCCACCCAAACCTGGTCCGGCTCGTGGGTTGCTGCCTGGAGACGGAGGTGCCCAT
GTTGGTCTATGAGTTCGTCCCCAGAGGGAGCCTTCAGGACGTGCTTCACGGAAACAAC
CACCCTCTCCCACTGGAAACACGCCTCGACATTGCTATCAGCTCCTCTGAGGGGCTCG
ATTACATGCACTCGCAGAGCCAGATGGTCCTCCACGGTGATGTCAAATCTGGCAACAT
CCTCATCGACGACAGCTTCACGCCCAAGGTGTCGGACTTTGGGACGTCCAGGCTCATG
TGCATCGACAAGGACCACACCAACTGGGTGGTCGGGAACAGCAGCTACATTGACCCC
GTGTACATGAAGACCGGGCTGCTCACCGCTAAGAGCGACGTGTACAGCTTTGGTATCG
TGCTACTGGAGCTCGTTACCCGAAAGAAGGCTAGGTACGGGGAGAATCGTAGCCTTCC
GATGGACTACGTCAAGGCTTCCAAGGATGGCACGGCAAGGCAGCTGTTTGATGAGGA
GGTTTCATCCAATGTTGAGGAGAACATGGAGTGCCTCGAGGACGTTGGCAAGATTGCA
GTGCAATGTCTCGAGGAAGACGTGAATAACAGGCCTACCATGGAGAAGTCAGGAAG
GAACTTGAAAAGTGTAAGACACAATGGTTGCGAAGCCAGGGGAGGGCAAATGAGGTA
ATCCCCTAG

>*WAK2*_02-5B-318, protein, 753 aa
MSITINSKRSSKLCYRNIPLWKKNLQTTMPSTERGAYESGPSGLRPSPLSMIGTRRPCVQSN
LAMTLRQTLLLLLVFHASLATTAEFGEPFPPPAPASPEPKNCPAKCGEIDIPYPFGVGAGCS
LSGRFVLTCNEMTSPPTLLTGTVKVASITLETAQMVVNTYLTYSCDVPTSNTTINHTSTDM
ALNVVTPFLLSPSENVFTAVGCSSTARIKGRGASPYLTGCITTCARVNDTGDDGTPCSGHG
CCQASLTPNLTQVSVEWENRGGRSPVAENLCQYAFVATKGWYSFRKNDLIGNMTFANRL
GSGPVVPVVLDWAVRDGTCPPTLEGNGKEIVPNGAACVSSESYCVNASHGAPGYFCNCSK
GYTDIDECALRRSPNSTMYKNIYPCRGGTCHDTEGDYECKCNFGRRGDGKSDKGCEPVLS
RPAVAVIGERENRKLRDRFNRNGGSFLKSAGIEIFTKDKLGRITNKYSCIIGKGAFGKVYKG
TTDTGVVVAVKRSIIVNEDRQKDFANEITVQSKISHPNLVRLVGCCLETEVPMLVYEFVPR
GSLQDVLHGNNHPLPLETRLDIAISSSEGLDYMHSQSQMVLHGDVKSGNILIDDSFTPKVS
DFGTSRLMCIDKDHTNWVVGNSSYIDPVYMKTGLLTAKSDVYSFGIVLLELVTRKKARYG
ENRSLPMDYVKASKDGTARQLFDEEVSSNVEENMECLEDVGKIAVQCLEEDVNNRPTMG
EVRKELEKCKTQWLRSQGRANEVIP

Supplementary Figure S2 Genomic sequence of *QFhb.mgb-2A* region isolated in the FBH-susceptible durum wheat cv. Saragolla (^{S}P), containing the *WAK2* gene. mRNA and protein sequence are also reported.

Grey box: exons. Sequence in the box: transposable MITE element (165 bp). Green box: 6 bp insertion. Yellow: SNP

> *QFhb.mgb-2A*_Saragolla, gDNA
ATGGATCAGGCACCAGCAACTAGCCGCTAAAATATGAGAGCCAGTAGTCGACATGAGTA
CTTTGGACATTAGGAAGACGTATGCGCCAAGCCCGAGCATCATCATAGCACGCCCGATG
CTGCAGGTCCAGCCAGCGCTCACAAAACACAACCCCATTGAGATGTTTCCAATGAGAGTC
GAGAAATCCAAGGACGCACACTTTCGAGAGCCGAAAAGATCTGTTGTCGGGGCGGTTTT
GACAAGTTGTGGTGGAAAGATCACATAGTCGCTATCCGCCTGCCGGCATTGCTTGGCGGC
GAGGGAAGAGGAGCTGGGGGAGGGAGGAGGGGGGCTAGTGTTTCCCAGAGCCATTTTA
GACTAGCCCATCGCAGTCTTCCATCAATTTCACTTATGATCTCTGCCGTTTCTAAAGAAAC
TCTCATCCAATGCACTGCATTATCGTCTGCTAATCCTTCAAGTTGAATTTTATCAATATAG
CATAGAATTACCGAGGAAATGAAACATGTGTTATTTTTTCTCTCGAATATGCACAACGTG
CGTATCATATTATATAAAACGAAGGCGGGGGTACAAAACCCACTACATCATTGATGTTAC
AATGTAACTACAACACCAACAACGACAATACAACCCATCTATCAGGAAACTACCACACA
TCCAATGGCTACTCTCTTCTCGCCCACCACAATAGGTCCACCAAGAGATCATCAGGTTCT
CCTTTTAACAATCCGGTCTGCTGCCAAGCTCTACCTTCGTCTGCTATCCTCGCGGCCACCA
AGTCAAGAGATGGGGATGCACCATCAAATACAACCACATTCCGATGTTTCCATAGCTCCC
ACAATGCAAGAGTGAAGATGGCCCGGATTTTCTTTGGATTTTACGGCTCGTTGGCCCTGT
CTACACACCATACCATTAGCATCCATTCTTGCCTGCTGAGAATTGTGCACAGCGTCCTCC
ATATGGTCCTTGCAAACACACAAGTCAGCAAAATGTGGTCAAGTGTGTCCTCCTCCTGTG
TACAGAACAGGCAGGTGTCCTGGTGTGCTAATCCTCGTCGGGCAAGTCGATCTGAGGTCC
AGCATATGTTCTCCATGGCGAGCCAAAAGAAGAAACAACATCGTAGCGGATCCCTCGAT
TTCCACGTGAATTCCACGGTTGGCACCACCTCTCTCCCTGCAAACTTGGCGGCGTATGCA
GATCTAACCGAAAACTGACTATCGTTTTCCCCTGACCACATAATGGTGTTTGTAGCTCCTT
CATCGAGTTCAACTATGTCCACCATTTGCCATACTCCCATGTACTCTTGTAGTGCGTCAAA
CTCCAAGTTTGGGCCAACATCCATCGTCCAAGTTTGGGCCAACGTCCATCGTCCAGGTTC
CCCTAGTCAATGCCTGTGGACAGTTCGAGATGTTTTGGCGCGCTGCGGGGTTCTAACATA
GAGCGCCGGTGCAAGTTCCTCTAAATGTGTGTTATGTTGTATATCATCTCTTCAGACAATC
CAACAAATAGGTAATTAAATTTATACGCGCTATCTAGTTAGACTCGCATTAACTTGACAA
AAGTAAGATTTAGCGTATTAATGCTTGGCCTATTAAAAAGATTTATACTAGTCCTTGAGA
TGGTGAGTATATAAAGTGGCATGTGGCAAAGTAATATACGCATAGTGAGGGATGACCAC
AGGAACTGGCCTAAGAAACACGCATATATGTCATCATATATATGTCATCTTGTTGAGCTC
ATGTCACCATAGCTTTGAAAGGGACACAAATTATGCATCGTTAATTATTGAGCTTGTGTG
CTAGCTCAGAGACAACCACGGGGAGTTTGGGACTAGCCTAATACACAAACATATATGTC
ATCGCACATACGTCATGTTATTGTGCTTATGTCACCACAATTTGGAAAGTGACACAGACT
GTGCGTCCTTATAATTAATTACTGGGTTTGTGTGCTAACCCATCCACCAACCATTCAATTT
GCATCAAATAAGTAAGTAATGATGTCAATTTGTCACACGGCCCATGTCGATCACCATCAA
CTCAAAAAGAAGCAGCAAACTATGTTATCGCAACATTCCTTTGTGGAAAAAGAATCTTCA
AACGACAATGCCATCAACAGAACGTGGAGCATATGAAAGTGAAACACAATCAAGGTATC
TTTTCTGGGTTTTTTTTAGGGCAAAAGTATCTTTTCTGGTGATTTGCCAACGCCACACTAT
CCAGGTGGGCCGTCAGGGCTACGACCATCACCTCTGTCGATGATTGGTACAGTAAGTATG
TTGAGCAAGATAAACGAGCATCAGGTCGACACAGCAGGGTGCCTGGCGAGTGGTGTGCA
ATAGAAGTCGTAGTTCGACCAAACTGTGGCCATGGGTCACTCAACTTTCATGTCAGTCAG
TTTGTGTTACTATATATATATGGGTTATGGGTATCAGAGGAGGCCGTGCGTCCAAAGCAA
TTTAGCCATGACACTGCGACAAACCCTCCTCCTCCTACTTGTCTTCCATGCCTCGCTCGCC

ACGACCGCCGAGTTTGGCGAGCCGTTTCCGCCCCCAGCCCCGGCGTCGCCTGAGCCAAAA
AACTGTCCTGCCAAGTGCGGGGAAATAGACATCCCCTACCCATTCGGCGTCGGCGCTGGA
TGCTCCCTGTCGGGCCGCTTCGTCCTCACCTGCAACGAGATGACGAGTCCTCCAACCCTG
CTCACCGGCACAGTCAAGGTCGCCAGCATCACGCTGGAGACGGCGCAGATGGTGGTCAA
CACCTACCTGACCTATAGCTGCGATGTGCCGACCAGCAACACTACGATCAACCACACGA
GCACCGATATGGCCCTTAACGTCGTCACCCCGTTCCTGCTCTCGCCATCCGAGAACGTGT
TCACGGCCGTCGGGTGCAGCTCGACGGCGAGGATCAAGGGCCGCGGCGCGAGCCCCTAC
CTCACTGGCTGCATCACGACCTGCGCCAGGGTGAAAGACACCGGTGACGACGGCACACC
CTGCAGCGGGCACGGCTGCTGCCAGGCCTCGCTGACTCCTAACCTCACCCAAGTCAGCGT
GGAGTGGGAGAACAGGGGAGGCAGAAGTCCTGTGGCTGAAAACCTGTGCCAGTATGCTT
TCGTCGCTACCAAAGGCTGGTACGTGAAGAAATTCCTTTTCCCCTTTTCCCCTTTCAAATC
ATCGTATACAGAATAACTTGGCAATTAACCCACCTTCCTATAACGTCCTAGTAAGGTCTA
TGGGTTTCACTTTCACTGAGATTTTGATGAATTTCATTCAATTTCAATAGACACAGAAATA
TTTACCTGTTGGTCATATTTCAGTGATTTGTGTTGTGCATAGGAATTCAAATATTTTTCTA
AATATTTTTGAACTTTTTCAAACATTTTTTTAATTCAAGTGAACATTTCGGTCCTTTGGCC
AAAATGCAAACCGAAATCACTGAACTTCAGTTATTTCAGTAGGTGCTAAACTGTTTCTGA
AACTGAAATTTAAAACCAACGAGTACTACTACTGATTATAACTTGGCAATTGCTGACAGA
ACATATCATCGAAACAAACATGTTTTTTCGAAAAGGAGGATTACCTCTGATCTTTGCATC
AAGCGATGTACACAATCATTTGTTTATTAAGGGAAACGATTCCCTACGACCGACCGACCG
AAGCTTTGGCCAGTCGGGTCGCTCCCGACCCACGCGTCGCACACCCACCAATGCTAGTGT
GCCACGCGTCCCTATGTTCTTGTGCAATGCTAAAAAGGCACCAAACGGCGACGGCTCCCA
GCACTGACCGGAGCGGGATGTTGCAACCGTCAAAAATACGAGAGCATACCTAAACACCT
ACCCTGTTATTTGACTGCCATCCAAATTGGTTGTATGTAGTACATGTTACCGTCTCACATC
GGTTGCATCCAAAGGCCATAAGTTCCCTCACGTCTGCATGGTGTAGTAATGACCACGTAT
GGATCGATTCAAACGATCGGTAGATAACCTGCAAAGAGTTTGTTGTAATGTTTGTTATGT
TAAACACACAATCATTCCGATAGTTGCAAATAGCCCAAAGTAATGCACAAGCTCTGACTC
GAATTCTTGCCTTTGTATTGGACTTAACCCTATCTAACTAGCTACTACCAAACCGGATTAG
GGATACTATTAGGTGGAGGTATGTTAAAGGACACATGTATTATTCGTCATAATAACTTGG
CTAGTGGGCATATAAGAAATAAGTGTTGAATCATTTTCTCGTGGTCACAGAAATAATGGT
GTTTATTCCCTTCCCAACTACGTTTTGCCAAATTGTACTTTGTTAGAATCACGCTCTTATG
AAAAAAACTCAAGAAAATCTTGATCTTTAAAGGCACTTTCGTTATCCAGAAATGAATAGA
CAGTGTGTTCCAGTATTAATCAAGTCGACATACATAGATTTAACGAAAAACAAGTCTGAA
ACAGAAAGAGCCCTTCGGAATGTATCGGGCTCATTGACAAGTTGACCTCCATCAAGCTTT
GCATTAAATGCAGCCAGTGATCCAATCTTTCCCCAATCAGTGTTCATCTGAACTGTACATT
TAGAGGAACGATCCCAATATTGTACTCACAGATTCGTCCTTATGATGTACGATATTATAA
AAGCGTTAGATAATACTATAAGGCTAGAGGTGTCTCTCCTAGCCAAATATCCTCCCAAAA
TCTAATTGTACGCCCATCTCCAATAATAAACTTGCCCCTATTAAAGAAGATGACCTTATTT
CGCATCAAAGCCTTCCAAAAGGGTGAATCGTTTGGCTAGGCCATTACCTGCGAGAAGGTC
TTGGAGTGTAAATGCTTGTTTCAAAACAACATGTAACCACTAACCATAGGATCTAGTTTA
GAAGATCTTGTCGCAACAAACCAATGGTGAGAAAAAAAGATCCTAGAACAGATTTATGG
TTGGTGCTTAATGTAAGAATATAGTGAATCTTAGTGATATCATGCTTGCACACGAGAGAG
ATGGGGTGGTGTTGCACGGTATGGTCAAGATGCATCGCAGCATCCTAGCCTGGTCCATCA
TTCTACCAAAGTTATAGGTTTTATGAAGATAATGGCATGTTTTTAGTCTAGACATATGTAC
AAACGCTTCAAGTTGCAACAATCTTCCCAAAAAAAAAGTTGCAACAATCTTCACATATACT
AATGGTTGAATTTGTCGAGCAGGTACAGTTTCAGAAAAAATGACCTGATTGGGAATATG
ACATTCGCCAATAGACTTGGAAGCGGCCCTGTTGTTCCAGTTGTTCTTGACTGGGCAGTT
AGGGATGGAACATGCCCCCCGACTCTGGAGGGTAACGGCAAGGAAATTGTTCCTAACGG
TGCCGCCTGTGTTAGCAGTGAAAGCTATTGTGTAAATGCAAGTCATGGAGCACCGGGCTA
TTTCTGCAATTGCTCCAAGGGATACACCGGTAATCCATACAAAAAAAAATGGGTGCACAA
GTATGTAAACTTCACTCCACTTGTTTGTTATTGCATATATATTCCTCTCGCCTCAGAGTAA
TTTGAGATCTTTTTAATTGTATAGCACACCCTGGATACCGAGGAGGACAATTTGACCTTG
GATAAGATTAGGGATCCCAGGATTTTTAGTGCTCGCTCACTATTTTTAACATTAATAAACT
TGGAAGTAAATAATAGTATGATACTAATGTAAGACTTACATATTTCATCCCTTGTGGCCT
TATTAATTCAAAGTCAGGATCTACTACAGCGGTACGGCCTTCCTTCTACAAAAATGATAT
TGATTGGTGTCTTTACTTTCATGCACTAAACTGAACAATGTGCAATGTTTTTCGTACATTT

TTCAGATATTGATGAGTGCGCATTACGACGATCACCAAATTCCACAATGTACAAGAATAT
CTATCCTTGCCGTGGAGGGACATGCCACGATACCGAAGGTGATTATGAGTGTAAGTGTAA
TTTCGGACGAAGAGGAGACGGCAAGAGTGACAAAGGTTGTGAACCCGTATTGTCCAGAC
CTGCAGTCGCAGTGATAGGTGAGGTATTGTATATATGCTCTACTTTCCTAAACTTCATGAC
AAAGCAGTCACCATTCCAACAACTTGCATCATATTCTTATTTCAAAAAAAAAACTTACAT
CATGTTCTTATATCACTGAATATCTATACCAAATTACTAGTACATGTCATCAGGTGAAGTT
GTGAACAGTTATGACTGTCTCTTAATATATACTCCCTCCGTTCCTAAATATTTGTCTTTTT
AGAGATTTCAAATGGACTACCACATACGGATGTATATAGACATATTTTAAACTGTAGATT
CACTCATTTTGCTCCGTATGTAGTCACTTGTTAAAATCTCTAGAAAGATAAATATTTAGG
AACGGAGGGAGTAGTTCGCATGACACCATTAACATGCATATTTTTCGAAAAGGAATGAA
AACCCTTGGCCCTGATGCACACAGCCTTTTGCATGAACATGCATGACCAAATCTTCTCTT
GGGTACATGTCATGCAGTTACTTGGAGCAAGCTAGTACATGTCATATGTATGAACTCTCA
TGTTAGTGCTCGATCTCTGGCCATCTTGAAAAAAAATGTAACTACTTATATATGTCATCTG
CTTGCAATTGCATGCAGGAACAATTGGCGCAATCGCATTACTGTTCGTGCTAGTAATATT
TTTGCACATGGAGCGAGAGAACAGGAAGCTGAGGGACCGATTCAACAGGAACGGCGGG
TCGTTCCTCAAGAGCGCCGGGATCGAGATCTTTACCAAGGACAAGCTGGGTCGCATCACA
AACAAGTACAGCTGCATCATTGGCAAAGGTGCCTTCGGTAAGGTCTACAAGGGGACCAC
CGACACCGGCGTTATTGTTGCTGTGAAGCGCTCCATCATCGTCAACGAGGACCGGCAGAA
GGATTTCGCGAATGAGATCACGGTCCAATCCAAGATCAGCCACCCAAACCTGGTCCGGCT
CGTGGGTTGCTGCCTGGAGACGGAGGTGCCCATGTTGGTCTACGAGTTCGTCCCCAGAGG
GAGCCTTCAGGACGTGCTTCACGGAAACAACCACCCTCTCCCACTGGAAACACGCCTCGA
CATTGCTATTAGCTCCTCTGAGGGGCTCGATTACATGCACTTGCAGAGCCAGATGGTCCT
CCACGGTGATGTCAAATCTGGCAACATCCTCATCGACGACAGCTTCACGCCCAAGGTGTC
GGACTTTGGGACGTCCAGGCTCATGTGCATCGACAAGGACCACACCAACTGGGTGGTCG
GGGACAGCAGCTACATTGACCCCGTGTACATGAAGACCGGGCTGCTCACCGCTAAGAGC
GATGTGTACAGCTTTGGTATCGTGCTACTGGAGCTCGTTACCCGAAAGAAGGCTAGGTAC
GGGGAGAAGCGTAGCCTTCCGATGGACTACGTCAAGGCTTCCAAGGATGGCACGGCAAG
GCAGCTGTTTGATGAGGAGGTTTCATCCAATGTTGAGGAGAACATGGAGTGCCTCGAGG
ACGTTGGCAAGATTGCAGTGCAATGTCTCGAGGAAGACGTGAATAACAGGCCTACCATG
GGAGAAGTCAGGAAGGAACTTGAAAAGTGTAAGACACAATGGTTGCGAAGCCAGGGGA
GGGCAAATGAGGTAATCCCCTAGTATCTTGTACTAGTCAAGTATAGCCAATTTCTTTTCGT
TGATGTAGTCTACTGCAGTTATGACTTGAATATGTTTTCTCCAGTGTGTGATGTACATGTG
CTTGCTATGCAATTGCAATAATTTATAGTGTGTGACTAAGGCTCAGTTTAATTACGGTTGC
GAGTTGTAAGAACTTCTTTGTGACTAAGCACCCTCAAGTCCCTGACAAATGTAGAAGCTG
ACACTACTAGAAAATGAGCTTCCTAATCTCCCGGCTGACATGGCGAAAATGAATTGCACT
CTTGATTACACATCAATGGAAAACTATCTTGAAATAGAAATTGACAGGATGAAACAGAT
GCGAGTGTATAGACATTCAAAATCAAAACATTTGGGTATGAAACTAGAGGTGGGAAGGG
AACACTAAGCTGGATGAATGCAGGAAATGGAGATGCTCTGTACGTGCGCCGTCCAGCTG
CTTCGTCTGCATTACAACAAGGACAATTAACAACTGAATCAGGTTCATACCATGCATACA
TGCATTAACCAGTTAAGAGTCAGCAGCAACTAGGGGCGTCTACCTGTATCTGCAGGTTCA
GCAAGTAGGGGCCTCTATGTGCCCGGGTTTCCTTTTCCTCGCTCCGGCATCTGAAATTCCC
TTCCTTGGCAAATGCGCCACAGACCACAGCTACGGAACAAGTGCAACTGATTGGTTGACC
ACACAGCCGCGGTCGCGCAATGCGCCGAGCACCTTGTGGGCGTGGTGGACATGCGGCCG
CCCGGCCTGGCTGCATCGCTGCTGGAGCAACGGTGTCGCGGTTCCGGGAAGGCAGATAA
CTAGCGAAGGTTGCCAAGGACGACGGCGATGAGCCGGCACAGGTGTGATGTGGCCAGAC
GGCGGAAGCCGGCGGCGGTCTGGGACGCCGGTGCGCGATGACAAGTCTCGCTAGAGGAC
AAGTCGGGCCACCGATGGCTTTGTGCAGCCCACGGCCATTTTGACTGGGCCGCCCATTTG
ACGAACACGGCGAGCTCCTCGTTGACAGCTACTGTAGCTTTGCAGTTTGTCTCAAAAAAA
GCTACTGTAGCGTGCTTGTCTCAAAAAAAAAATCTACTGTAGCGTACGAAGTACTATAGCT
CGCAGGTGCACTGTAGCTTAAAAACAAAAACTTGCAGGTTTTACTGTAGCTGCGTTCAAA
AAATGCAACCAACTTTTGAAAAAGTGGACTGTTTGATATACAAACAATTTTTCGAAATTT
TACAACAAGTTTTGAATTTCGAATATTTCTTGAATACTTTGAACAATATTTGAATTTTTTG
CAATATTTTTGCAAAACACGAAATTTTTTGGAAAACATTATCAAGTTTTTTGAAATTCTGA
AGCATTTTTGAATATTTTTATTAATTTTTTAGAAAAGTGAACATTTTAAAGGAAAGCATGA

ACCAAAATTTGAAATTTGTAGCATTTTATGGAAAGCGAACATTTTCCAAAATTCTGAACA
AGTTTTGAAGGCATTTTTCAAGAATTTATGAAAATAAAAAAGACAAATAGACGCAAAAA
AAACATAAAAGGAAAAAAAAGGGAAACCGAAACAAAAATGGAAAACTAGAAAAAGCA
GTAAAGAAAATGACAGAAAAAAGAAAACCGGTTCAGGATCCTTCTAAAAGATTCTCAAA
ACCGATTGAAAAATATCCTCTCCTTCATCCCTTTTCTCTGCGGCAAACACGGTCTCCCTCC
CCTCTCTCAGCGCGCGCGCCACACCATCCCCTCGAGGCTTTGACAGGGTGAGGGGTGGTT
TCAGGCTTCGTTCTTATGCGGTTGGTGGTTGGCGTGGCGTTGTAGCTAGGTGAGTATTGG
ATCGCATCTCTAATCTGTCTCACCCATTTTTGTAGGTCAACAACCCGAACTTCCTACACTG
TACTATGTATACACATGCTAATACTTCTTCTGTGTTGGAGGTACTTGCTCTTCTTGCTTGCT
AGGATGATAGGATGATGCCATGCATGTGAGATACCAATTAAGAAGGGCAATAATTTACT
ACTACTTGTTATTATGTGTGCTAAATATATGGCATGCCAAACTATATCAGCCAATCATCA
GCCTTGTAAGTTGTAGCGTTAAATAGATTATATTATTTGCAGGGCCTTTTTTTGGGAAAAA
TATAACCTATGCGGCATCAGTCAACGCTTCATTTGGCTGTTTCAGACTGGCCTGTGTTAAT
CCACTTATCCAAATGTGCACAAAAATAAAAAATCATACTTACAACCCAACTGACACTCCT
CTCCATACTTTAAGGGCCTCCAATCCATTTTACTCTTCTTTATTAGATACGGCTGGGCTCT
AATACACACGGAATATTTGAGTTCTCAAAAGTTTGCCTTCTCTCGACAGAAGATGTTCCA
AGGTCTTAAGTGGCTAAATCTTGCCTTTTGCAATTAGAGATATGGCATGATGTGTGCTAC
GCTGATATTTTTTGTTGAATGATCCTTTGTAATCTTGCTACTCTAACTTTCTTGTTTCGCTG
ACTCTATTACGGCAAAATAAACCAAGGTTGTTTCTTGACTGTCTTGTGTATGAAGAAACA
TCAACAATGATAACAAAATATGGATGGGTGCAGATACGATTGACGGTATAAAGTTTGAA
CTCTCATTATGC

>*WAK2*_Saragolla, mRNA_1, 1278 bp
ATGTCGATCACCATCAACTCAAAAAGAAGCAGCAAACTATGTTATCGCAACATTCCTTTG
TGGAAAAAGAATCTTCAAACGACAATGCCATCAACAGAACGTGGAGCATATGAAAGTGA
AACACAATCAAGGTGGGCCGTCAGGGCTACGACCATCACCTCTGTCGATGATTGGTACAT
CAGTTTGTGTTACTATATATATATGGGTTATGGGTATCAGAGGAGGCCGTGCGTCCAAAG
CAATTTAGCCATGACACTGCGACAAACCCTCCTCCTCCTACTTGTCTTCCATGCCTCGCTC
GCCACGACCGCCGAGTTTGGCGAGCCGTTTCCGCCCCCAGCCCCGGCGTCGCCTGAGCCA
AAAAACTGTCCTGCCAAGTGCGGGGAAATAGACATCCCCTACCCATTCGGCGTCGGCGCT
GGATGCTCCCTGTCGGGCCGCTTCGTCCTCACCTGCAACGAGATGACGAGTCCTCCAACC
CTGCTCACCGGCACAGTCAAGGTCGCCAGCATCACGCTGGAGACGGCGCAGATGGTGGT
CAACACCTACCTGACCTATAGCTGCGATGTGCCGACCAGCAACACTACGATCAACCACAC
GAGCACCGATATGGCCCTTAACGTCGTCACCCCGTTCCTGCTCTCGCCATCCGAGAACGT
GTTCACGGCCGTCGGGTGCAGCTCGACGGCGAGGATCAAGGGCCGCGGCGCGAGCCCCT
ACCTCACTGGCTGCATCACGACCTGCGCCAGGGTGAAAGACACCGGTGACGACGGCACA
CCCTGCAGCGGGCACGGCTGCTGCCAGGCCTCGCTGACTCCTAACCTCACCCAAGTCAGC
GTGGAGTGGGAGAACAGGGGAGGCAGAAGTCCTGTGGCTGAAAACCTGTGCCAGTATGC
TTTCGTCGCTACCAAAGGCTGGTACAGTTTCAGAAAAAATGACCTGATTGGGAATATGAC
ATTCGCCAATAGACTTGGAAGCGGCCCTGTTGTTCCAGTTGTTCTTGACTGGGCAGTTAG
GGATGGAACATGCCCCCCGACTCTGGAGGGTAACGGCAAGGAAATTGTTCCTAACGGTG
CCGCCTGTGTTAGCAGTGAAAGCTATTGTGTAAATGCAAGTCATGGAGCACCGGGCTATT
TCTGCAATTGCTCCAAGGGATACACCGATATTGATGAGTGCGCATTACGACGATCACCAA
ATTCCACAATGTACAAGAATATCTATCCTTGCCGTGGAGGGACATGCCACGATACCGAAG
GTGATTATGAGTTCACTTGTTAA

>*WAK2*_Saragolla, protein_1, 425 aa
TINSKRSSKLCYRNIPLWKKNLQTTMPSTERGAYESETQSRWAVRATTITSVDDWYISLCYYI
YMGYGYQRRPCVQSNLAMTLRQTLLLLLVFHASLATTAEFGEPFPPPAPASPEPKNCPAKCG
EIDIPYPFGVGAGCSLSGRFVLTCNEMTSPPTLLTGTVKVASITLETAQMVVNTYLTYSCDVPT
SNTTINHTSTDMALNVVTPFLLSPSENVFTAVGCSSTARIKGRGASPYLTGCITTCARVKDTG
DDGTPCSGHGCCQASLTPNLTQVSVEWENRGGRSPVAENLCQYAFVATKGWYSFRKNDLIG
NMTFANRLGSGPVVPVVLDWAVRDGTCPPTLEGNGKEIVPNGAACVSSESYCVNASHGAPG
YFCNCSKGYTDIDECALRRSPNSTMYKNIYPCRGGTCHDTEGDYEFTC

>*WAK2*_Saragolla, mRNA_2, 966 bp
ATGGAGCGAGAGAACAGGAAGCTGAGGGACCGATTCAACAGGAACGGCGGGTCGTTCCT
CAAGAGCGCCGGGATCGAGATCTTTACCAAGGACAAGCTGGGTCGCATCACAAACAAGT
ACAGCTGCATCATTGGCAAAGGTGCCTTCGGTAAGGTCTACAAGGGGACCACCGACACC
GGCGTTATTGTTGCTGTGAAGCGCTCCATCATCGTCAACGAGGACCGGCAGAAGGATTTC
GCGAATGAGATCACGGTCCAATCCAAGATCAGCCACCCAAACCTGGTCCGGCTCGTGGG
TTGCTGCCTGGAGACGGAGGTGCCCATGTTGGTCTACGAGTTCGTCCCCAGAGGGAGCCT
TCAGGACGTGCTTCACGGAAACAACCACCCTCTCCCACTGGAAACACGCCTCGACATTGC
TATTAGCTCCTCTGAGGGGCTCGATTACATGCACTTGCAGAGCCAGATGGTCCTCCACGG
TGATGTCAAATCTGGCAACATCCTCATCGACGACAGCTTCACGCCCAAGGTGTCGGACTT
TGGGACGTCCAGGCTCATGTGCATCGACAAGGACCACACCAACTGGGTGGTCGGGGACA
GCAGCTACATTGACCCCGTGTACATGAAGACCGGGCTGCTCACCGCTAAGAGCGATGTGT
ACAGCTTTGGTATCGTGCTACTGGAGCTCGTTACCCGAAAGAAGGCTAGGTACGGGGAG
AAGCGTAGCCTTCCGATGGACTACGTCAAGGCTTCCAAGGATGGCACGGCAAGGCAGCT
GTTTGATGAGGAGGTTTCATCCAATGTTGAGGAGAACATGGAGTGCCTCGAGGACGTTG
GCAAGATTGCAGTGCAATGTCTCGAGGAAGACGTGAATAACAGGCCTACCATGGGAGAA
GTCAGGAAGGAACTTGAAAAGTGTAAGACACAATGGTTGCGAAGCCAGGGGAGGGCAA
ATGAGGTAATCCCCTAG

>*WAK2*_Saragolla, protein_2, 321 aa
MERENRKLRDRFNRNGGSFLKSAGIEIFTKDKLGRITNKYSCIIGKGAFGKVYKGTTDTGVIV
AVKRSIIVNEDRQKDFANEITVQSKISHPNLVRLVGCCLETEVPMLVYEFVPRGSLQDVLHGN
NHPLPLETRLDIAISSSEGLDYMHLQSQMVLHGDVKSGNILIDDSFTPKVSDFGTSRLMCIDKD
HTNWVVGDSSYIDPVYMKTGLLTAKSDVYSFGIVLLELVTRKKARYGEKRSLPMDYVKASK
DGTARQLFDEEVSSNVEENMECLEDVGKIAVQCLEEDVNNRPTMGEVRKELEKCKTQWLRS
QGRANEVI

Supplementary Table S1 Identification of candidate genes within the peak region of *QFhb.mgb-2A* comprised between IWB5988 and IWA5087 (5.3 cM). Bold: most associated SNPs to *QFhb.mgb-2A* QTL and *WAK2* genes.

Wheat region	Mapped SNP	Functional annotation	Expression in spike (Zadoks 65, FPKM)
Traes_2AS_1D11E82B5	IWA2059, IWB30909, IWB48972, IWB10663	FAR1 Protein	19.73
Traes_2AS_B5FF1129E		GDT1-like protein 3	7.88
Traes_2AS_1A5627411	IWB5988	Arginine decarboxylase protein (ADC)	6.84
Traes_2AS_F239E74C6		Ubiquitin thioesterase otubain protein (OTUB), Alanyl-tRNA synthetase protein (AARS)	5.99
Traes_2AS_B8F717B57		F-box domain containing protein	5.23
Traes_2AS_CBB2430AD		Cytochrome P450, FAR1 Protein	4.72
Traes_2AS_28FF132E0		Flavin-containing monooxygenase 1 (FMO1)	3.66
Traes_2AS_66EBD8FC7		Expansin-A1 protein (EXPA1)	3.31
Traes_2AS_77FC0C7A4	IWB32072	SWI/SNF-related matrix-associated actin-dependent regulator of chromatin subfamily A member 3 (SMARCA3)	1.50
Traes_2AS_845E28901	IWB23172	Ubiquitin thioesterase otubain protein (OTUB)	1.21
Traes_2AS_3C22C11BA		Ubiquitin thioesterase otubain protein (OTUB), NADH-ubiquinone oxidoreductase chain 5	1.20
Traes_2AS_01DDBF899		UDP-glycosyltransferase	0.80
Traes_2AS_4ABCC7848		F-box domain containing protein	0.71
Traes_2AS_744F6C927		Germin-like protein 8-14, ethylene-responsive transcription factor protein	0.70
Traes_2AS_2B95E681C		L-ascorbate peroxidase 3, **Wall-associated receptor kinase 2 protein**	0.57
Traes_2AS_7F573BF04		Leucine-rich repeat receptor-like protein kinase family protein	0.44
Traes_2AS_2028486B1		Biosynthetic arginine decarboxylase	0.25
Traes_2AS_362BBDD74		5'-3' exoribonuclease 3	0.06
Traes_2AS_B5A6B1C99	**IWB56555, IWB63138**	**Wall-associated receptor kinase 2 protein**	0.05
Traes_2AS_440705688		**Wall-associated receptor kinase 2 protein**	0.00
Traes_2AS_A2393F908		Disease resistance protein RPP13	0.00
Traes_2AS_534751FCA		Disease resistance protein RPM1	0.00
Traes_2AS_1C1F32275		**Wall-associated receptor kinase 2-like**	0.00
Traes_2AS_C47F28085		Flavin-containing monooxygenase 1	0.00
Traes_2AS_76A7AED3C		Alcohol dehydrogenase 1	0.00
Traes_2AS_92A33FAC3		UDP-glycosyltransferase 74E1	0.00
Traes_2AS_DEC213280		High-affinity nitrate transporter 2.1 protein	0.00
Traes_2AS_6EFE41153		UPI000234F155 related cluster n=1	0.00
Traes_2AS_F7094CC33		Nitrate transporter 2 protein	0.00
